# Tick Immune System: What Is Known, the Interconnections, the Gaps, and the Challenges

**DOI:** 10.3389/fimmu.2021.628054

**Published:** 2021-03-02

**Authors:** Andréa C. Fogaça, Géssica Sousa, Daniel B. Pavanelo, Eliane Esteves, Larissa A. Martins, Veronika Urbanová, Petr Kopáček, Sirlei Daffre

**Affiliations:** ^1^Department of Parasitology, Institute of Biomedical Sciences, University of São Paulo, São Paulo, Brazil; ^2^Institute of Parasitology, Biology Centre, Czech Academy of Sciences, Ceske Budejovice, Czechia; ^3^Laboratory of Bacteriology, Tick-Pathogen Transmission Unit, National Institute of Allergy and Infectious Diseases, Hamilton, MT, United States

**Keywords:** cell-mediated immunity, immune signaling pathway, immune system, microbiota, tick-borne pathogen

## Abstract

Ticks are ectoparasitic arthropods that necessarily feed on the blood of their vertebrate hosts. The success of blood acquisition depends on the pharmacological properties of tick saliva, which is injected into the host during tick feeding. Saliva is also used as a vehicle by several types of pathogens to be transmitted to the host, making ticks versatile vectors of several diseases for humans and other animals. When a tick feeds on an infected host, the pathogen reaches the gut of the tick and must migrate to its salivary glands *via* hemolymph to be successfully transmitted to a subsequent host during the next stage of feeding. In addition, some pathogens can colonize the ovaries of the tick and be transovarially transmitted to progeny. The tick immune system, as well as the immune system of other invertebrates, is more rudimentary than the immune system of vertebrates, presenting only innate immune responses. Although simpler, the large number of tick species evidences the efficiency of their immune system. The factors of their immune system act in each tick organ that interacts with pathogens; therefore, these factors are potential targets for the development of new strategies for the control of ticks and tick-borne diseases. The objective of this review is to present the prevailing knowledge on the tick immune system and to discuss the challenges of studying tick immunity, especially regarding the gaps and interconnections. To this end, we use a comparative approach of the tick immune system with the immune system of other invertebrates, focusing on various components of humoral and cellular immunity, such as signaling pathways, antimicrobial peptides, redox metabolism, complement-like molecules and regulated cell death. In addition, the role of tick microbiota in vector competence is also discussed.

## Introduction

Ticks (Acari: Ixodida) are ectoparasitic arthropods that obligatorily feed on the blood of a diverse list of vertebrate hosts, including mammals, birds, reptiles, and even amphibians. More than 950 tick species have been described to date, which, according to morphological and physiological characteristics, are divided into two main families, Ixodidae (hard ticks), comprising more than 75% of tick species, and Argasidae (soft ticks); a third family, known as Nuttalliellidae, is monospecific (1, 2). As a result of blood spoliation [a single ixodid adult female can ingest more than ~1 mL of blood (3)], the host can suffer from anemia, which negatively impacts the productivity of livestock and causes a huge economic burden worldwide. For example, the estimated annual losses due to reductions in weight gain and milk production caused by the cattle tick *Rhipicephalus microplus* are approximately 3.24 billion dollars in Brazil alone (4).

In addition to ingesting blood, ticks also secrete saliva into the host during feeding. Tick saliva, produced by their salivary glands, returns excess water and ions to the host, thereby concentrating the blood meal (5). Tick saliva contains an arsenal of bioactive molecules that modulate host hemostasis and immune reactions, thus enabling blood acquisition (6, 7). The antihemostatic and immunomodulatory properties of saliva can also facilitate the infection of pathogens that use saliva as a vehicle to be transmitted to the host during tick blood feeding (6, 8). Indeed, ticks are versatile vectors of viruses, bacteria, protozoans and nematodes, which cause life-threatening diseases to humans as well as to other animals, including livestock, pets, and wildlife (9). Among human diseases, we highlight Lyme disease, the most common tick-borne zoonosis, which is caused by spirochetes from the *Borrelia burgdorferi* sensu lato complex. After transmission by the bite of an infected tick, the typical clinical sign of Lyme disease is erythema migrans, but infection can spread and affect joints, heart, and the nervous system (10).

**Figure 1:**
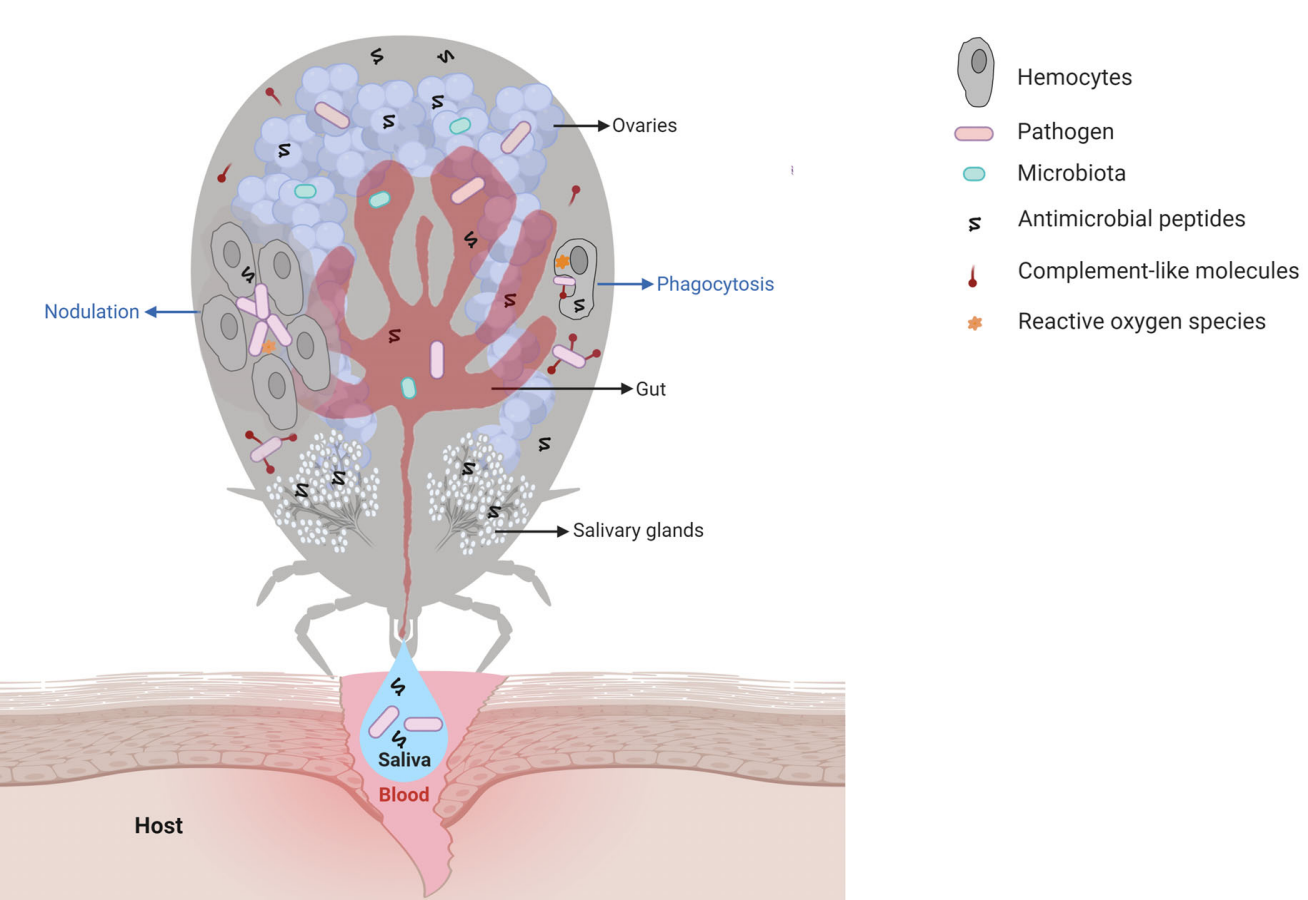
Main interactions among tick immune system components, microbiota, and pathogens. Pathogens ingested within the blood meal initially reach the tick gut, where they interact with components of the gut microbiota and with cytotoxic molecules, such as AMPs (hemocidins and endogenous AMPs) and possibly with factors of redox metabolism, despite not being fully comprised. Pathogens must colonize and/or cross the gut epithelium to reach the hemocoel, which is filled with hemolymph. In hemolymph, complement-like molecules attach to pathogens that can be engulfed or trapped by hemocyte-mediated processes named phagocytosis and nodulation, respectively. Invaders can also be killed by several types of effector molecules, including AMPs, complement-like molecules, and factors of redox metabolism. The tick salivary glands return excess water and ions from the blood meal to the host through saliva, which also contains antihemostatic and immunomodulatory molecules. Pathogens use tick saliva as a vehicle to be transmitted to the host, in which infection can be facilitated by saliva properties. Some pathogens can also colonize the tick ovaries and are transmitted to progeny. In the tick salivary glands and ovaries, as in the gut, pathogens must deal with the members of resident microbiota as well as tick immune reactions. Additional studies are required to elucidate the molecules responsible for hemolymph clotting and melanization in ticks.

The first organ that a pathogen acquired within the blood meal interacts with is the tick gut (**Figure 1**). Then, the pathogen must colonize the gut epithelial cells and/or cross the gut epithelium to enter the hemocoel, an open body cavity filled with hemolymph, the fluid that irrigates all the tissues and organs in the tick. The pathogen must then reach the salivary glands. In each of these organs, the pathogen must counteract tick immune factors to be successfully transmitted through saliva to the vertebrate host in a subsequent blood-feeding (11). Some pathogens also have the ability to invade tick ovaries and can therefore be transovarially transmitted to progeny (**Figure 1**). Thus, elucidation of the immune factors involved in the interactions between ticks and tick-borne pathogens (TBPs) in each of these steps is essential to understand the biology of tick-transmitted diseases and may help to identify targets for the development of new strategies to block pathogen transmission. In this review, we present an update on humoral and cellular tick immunity components (**Figure 1**), including signaling pathways, antimicrobial peptides (AMPs), redox metabolism, complement-like proteins, and regulated cell death. Using a comparative approach with the immune system of other invertebrates, we highlight the challenges of studying tick immunity, the gaps, such as prophenoloxidase (PPO) and coagulation cascades, and the interconnections, such as immune system signaling pathway crosstalk. In addition, the role of tick microbiota in vector competence is also discussed.

## A Brief History of Studies on the Immune System of Arthropods

The first records of studies on arthropod disease date to the 19^th^ century, when Louis Pasteur investigated the cause of brown dots on the cuticle of larvae of *Bombyx mori* that predestined larvae to death and affected silk production in France (12). In the 1980s, the isolation of several immune factors from the hemolymph of arthropods that have a large volume of hemolymph, such as larvae of dipteran and lepidopteran insects, horseshoe crabs and crayfish, was achieved. Indeed, the first animal AMP to be characterized was cecropin, isolated from the hemolymph of the moth *Hyalophora cecropia* (13). After that, AMPs were identified as important effectors of mammalian immunity (14, 15). In addition to AMPs, components of the PPO cascade from the hemolymph of *H. cecropia* (16), *B. mori* (17), and the crayfish *Pacifastacus leniusculus* (18) were also elucidated. Some years later, the components of the coagulation cascade, another important arthropod immune reaction, were characterized in *P. leniusculus* (19) and horseshoe crabs (20).

In the 1990s, relevant studies on the immune pathways that regulate AMP production were conducted using the fruit fly *Drosophila melanogaster* (hereafter referred to as *Drosophila*) as a model (21–24). Among them, we highlight the identification of a kappa B (κB)-binding region in the promotor region of certain insect AMP genes (25) and the identification of Toll receptors, posteriorly identified to be homologous to interleukin-1 receptor of mammals (26). Some years later, with the improvement of molecular techniques and funding by major support agencies, such as the MacArthur Foundation, the World Health Organization, and the National Institutes of Health (USA), studies on the arthropod immune system were redirected to vectors of human diseases, principally mosquitoes (27). In this period, Sanger-based technology was largely used to elucidate genomes and generate datasets of expressed sequence tags (ESTs). After the development of next-generation sequencing (NGS) technologies, additional information on arthropod genomes and transcriptomes was added to public databases (28). Indeed, currently, more than 40 arthropod genomes are available in the VectorBase database (https://www.vectorbase.org/organisms).

Knowledge of vector genomes and ESTs allowed *in silico* comparisons of immune factors among species [for example, see (29–33)]. Moreover, studies with diverse approaches, such as transcriptomics, proteomics, and metabolomics analyses, to assess the arthropod response to different microbial stimuli were significantly expanded in the postgenomic era (28). The development and application of RNA interference (RNAi) and CRISPR-Cas9 technologies to arthropods [(34, 35), respectively] were also important to determine the role played by immune factors in the interaction between vectors and vector-borne pathogens.

Despite the importance of ticks as disease vectors, studies on their genomes and the molecular factors involved in their interactions with pathogens are scarce compared to studies on other arthropod vectors. The large size of tick genomes and the high contents of repetitive regions make genome assembly difficult. Indeed, the size of tick genomes is approximately 1.3 Gbp in argasids and 2.6 Gbp in ixodids (36). However, the genome of the cattle tick *R. microplus* is even larger and has been estimated to be approximately 7.1 Gpb, which is more than twice the size of the human genome (37). In addition, approximately 70% of the tick genome includes repetitive regions (37, 38). For this reason, until very recently, only the genome of the tick *Ixodes scapularis* had been annotated (38). Additional genomes were recently assembled by the use of NGS (37, 39). The scarcity of studies on the molecular factors involved in ticks and TBPs is in part due to the need for sophisticated structures to raise vertebrate animals to feed ticks, which is laborious and involves ethical concerns. In the last few years, artificial feeding systems have been successfully used to maintain laboratory tick colonies; however, an animal blood source is still required (40). Finally, the development of continuous cell lines derived from tick embryos, despite representing a mixture of different cell types, has also contributed considerably to studies on tick biology and their interactions with TBPs (41).

## Tick Immune Signaling Pathways

Blood feeding represents a challenge for hematophagous arthropods due to the large diversity of pathogens to which these animals are exposed. In contrast to other arthropods, hard ticks are strictly hematophagous, feeding on the blood of their host for several days. In addition, some species feed on a different host in each developmental stage (larvae, nymphs, and adults), thereby increasing the chance of either acquiring or transmitting pathogens. Therefore, ticks are important vectors of a large list of disease-causing pathogens (42). In addition to host pathogens, ticks are in close contact with the microbiota of the host skin, which may also be acquired within the blood meal (43). Ticks are also exposed to microorganisms in the environment during the nonparasitic phases of their life cycle. Hence, the immune system of ticks must be activated continuously to protect them from harmful infections.

Most of our knowledge on arthropod immune responses has come from studies on dipteran insects, especially *Drosophila* and the mosquitoes *Aedes* spp. and *Anopheles* spp. In *Drosophila*, invading microorganisms are mainly recognized by the Toll, immune deficiency (IMD), Jun-N-terminal kinase (JNK), Janus kinase/signal transducer and activator of transcription (JAK/STAT) and/or RNAi pathways (44). Nonetheless, the hypothesis that the level of conservation of arthropod immune responses might be high has been rejected by several studies on ticks, mites, lice, hemipterans, and others, and it is now recognized that the immune system displays remarkable diversification across the Arthropoda phylum (30). Tick immunity is, however, still greatly neglected and unexplored (45). Hence, we review the prevailing knowledge on tick immune signaling pathways alongside the connections between them and other equally important factors, such as AMPs, redox metabolism, complement-like proteins, and regulated cell death.

### Nuclear Factor-Kappa B Signaling Pathways: Molecular Regulators for Pathogen Recognition

#### The Unexplored Toll Pathway

**Figure 2:**
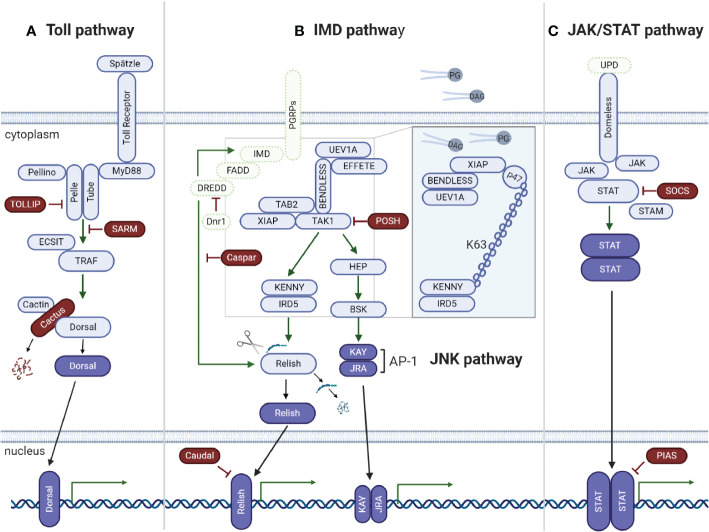
Tick signaling-related genes in the three main immune signaling pathways of arthropods: **(A)** Toll, **(B)** IMD, and **(C)** JAK/STAT. **(A)** A previous *in silico* study (31) showed that components of the Toll signaling pathway of arthropods are conserved in ticks: extracellular cytokine Spatzle (Spz), transmembrane cytokine receptor Toll, Toll-interacting protein (TOLLIP), adaptor protein MyD88, kinases Tube (interleukin-1 receptor-associated kinase 4 or IRAK4), Pelle (interleukin-1 receptor-associated kinase 1 or IRAK1), Pelle-interacting protein Pellino, TNF receptor associated factors (TRAFs), evolutionarily conserved signaling intermediate in toll pathway (ECSIT), sterile alpha- and armadillo-motif-containing protein (SARM), Rel/NF-kappa B transcription factor Dorsal, Dorsal inhibitor protein IkappaB Cactus (IkB), and interacting protein Cactin of the IkB. **(B)** Regarding the IMD pathway, genes encoding downstream members of both the NF-kB/Relish and Jun N-terminal kinase (JNK) branches were identified: peptidoglycan recognition proteins (PGRPs), enzymes involved in ubiquitination (UEV1a, Effete/Ubc13 and Bendless/Ubc5), X-linked inhibitor apoptosis protein (XIAP), negative regulators Caspar (Fas-associating factor 1) and POSH (E3 ligase Plenty of SH3), transforming growth factor-beta activated kinase 1 (TAK1), TAK1-binding protein 2 (TAB2), IRD5 and Kenny/NEMO (IKK*γ*), and Relish-like Rel/NF-kB transcription factor. The adaptor protein IMD (immune deficiency), its associated molecule FAAD (Fas associated protein with death domain), the caspase DREDD (death related ced-3/Nedd2-like) and Dnr1 (defense repressor 1) have not yet been described in ticks. Components of the JNK branch of the tick IMD pathway include mitogen-activated protein (MAP) kinase hemipterous (HEP), Jun-kinase basket (BSK), activator protein 1 (AP-1) transcription factors JRA (Jun-related antigen) and KAY (Fos-related antigen, Kayak). Some IMD pathway components were functionally characterized by (48) (Insert). The authors showed that the IMD pathway is activated by PODAG (1-palmitoyl-2-oleoyl diacylglycerol) or POPG (1-palmitoyl-2-oleoyl-sn-glycero-3-phosphoglycerol). Once activated, XIAP interacts with the heterodimer Bendless : UEV1a, leading to the ubiquitination of p47 in a K63-dependent manner. Ubiquitylated p47 connects to Kenny (also named NEMO) and induces the phosphorylation of IRD5 and Relish. **(C)** Components of the Janus kinase/signal transducer and activator of transcription (JAK/STAT) signaling pathway are also conserved in ticks: the transmembrane cytokine receptor Domeless, tyrosine kinase JAK (Hopscotch), transcription factor STAT, signal transducing adaptor molecule (STAM) and the inhibitor proteins PIAS (protein inhibitor of activated STAT) and SOCS (suppressor of cytokine signaling). The ligand of the Domeless receptor (UPD gene) was not identified in ticks **(C)**. Activated transcription factors are represented in dark blue; the immune signaling pathway components not yet described in ticks are represented in green.

The Toll signaling pathway is well studied in *Drosophila*, in which it is preferentially activated in the presence of bacterial [by recognition of lysine-type peptidoglycan (PGN) from the cell wall of Gram-positive bacteria) and fungal (by recognition of (1,3)-glucan polymers of D-glucose from the cell wall] pathogen-associated molecular patterns (PAMPs) (44, 46). *In silico* and genomic analyses have shown that ticks encode most Toll pathway components (31, 33, 38, 47) (**Figure 2A**), including the NF-κB Dorsal, indicating that conserved mechanisms of Toll pathway activation may exist. Indeed, the NF-κB transcription factor dorsal-related immunity factor (DIF) is the only component of the Toll pathway not yet reported in any tick species.

**Figure 3:**
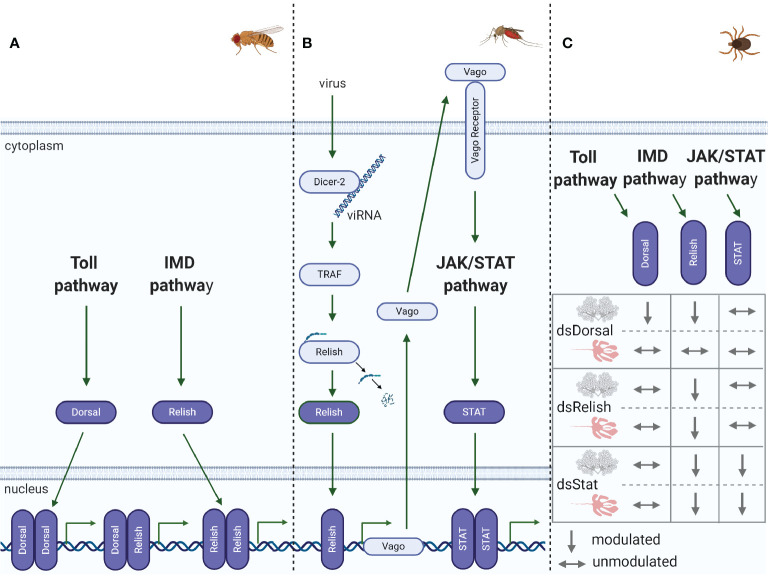
Immune pathway crosstalk. **(A)** In *Drosophila*, the DIF-Relish heterodimer activates the expression of both Toll and IMD pathway effectors, resulting in a stronger response against infection (50). **(B)** In *Culex*, after recognition of West Nile virus (WNV) dsRNA by Dcr-2, TNF receptor-associated factor (TRAF) stimulates Relish, upregulating Vago expression (51, 52). Vago is then secreted by the infected cell and activates the JAK/STAT pathway in adjacent cells, upregulating the expression of antiviral genes. **(C)** In *R. microplus*, knockdown of Dorsal downregulates both Dorsal and Relish expression in salivary glands, while the levels of all the transcription factors remain unaltered in the gut. Relish is also downregulated in the gut and salivary glands of STAT-deficient ticks. Conversely, knockdown of Relish results in the specific silencing of its target gene in both the gut and salivary glands (49).

How the tick Toll pathway operates is largely unclear. Rosa and collaborators showed that the Toll pathway components are differentially expressed in the tick cell line BME26, which is derived from the tick *R. microplus*, in response to live *Anaplasma marginale* and *Rickettsia rickettsii* (two obligate intracellular bacteria) and heat-killed *Saccharomyces cerevisiae* (yeast)*, Enterobacter cloacae* (Gram-negative bacterium) and *Micrococcus luteus* (Gram-positive bacterium) (31). Interestingly, heat-killed microorganisms upregulated the gene expression of the majority of the Toll pathway components, *R. rickettsii* upregulated some Toll pathway components and downregulated others, and infection with *A*. *marginale* (a pathogen naturally transmitted by *R. microplus*) downregulated most of the Toll pathway components. These results suggest that *A. marginale* may downregulate Toll pathway components in an attempt to favor vector colonization, which might correspond to coevolutionary adaptation. Of note, similar results were found for the IMD, JNK, and JAK/STAT signaling pathways (31). However, studies on the mechanisms used by this pathogen to overcome tick immune responses are warranted to confirm the authors’ hypothesis. In adult *R. microplus*, only Dorsal was downregulated in both the gut and salivary glands of *A. marginale*-infected ticks, while Relish and STAT remained unmodulated (49). Moreover, Dorsal silencing promoted an increase in the *A. marginale* burden as well as knockdown of Relish and STAT. However, while Relish dsRNA (dsRelish) specifically silenced Relish, this transcription factor was also downregulated in both the dsDorsal and dsSTAT groups, which might explain the increase in the *A. marginale* load in these two groups as well. To determine the pathway responsible for infection control, the gene expression of specific effectors of each immune signaling pathway, which are currently unknown, is warranted. As Dorsal-, Relish-, and STAT-encoding genes do not exhibit significant sequence similarity, the authors suggested the existence of putative crosstalk among the Toll, IMD, and JAK/STAT signaling pathways (49) (**Figure 3C**). Nonetheless, an off-target effect cannot be ruled out. It is also possible that the knockdown of immune signaling transcription factors exerts an effect on the gut microbiota, which, in turn, may modulate their gene expression. In contrast to the results obtained with *R. microplus*, gene silencing of Toll (ISCW018193) did not exhibit any effect on the *Anaplasma phagocytophilum* burden in the salivary glands of *I. scapularis* nymphs (53). However, gut colonization was not evaluated; therefore, it is not possible to guarantee that the Toll pathway is not involved in controlling *A. phagocytophilum* infection in this tick species. In a study carried out with *I. ricinus* cells (IRE/CTVM20), it was shown that the expression of a *Toll* gene (homologous to the *Toll* ISCW022740 of *I. scapularis*) is upregulated after 72 and 120 h of infection with flaviviruses [tick-borne encephalitis virus (TBEV) and louping ill virus (LIV)] but remained unmodulated in response to *A. phagocytophilum* (54). Conversely, infection with these flaviviruses downregulated the expression of three other Toll transcripts (homologous to ISCW017724; ISCW007727; ISCW007724 of *I. scapularis*), while Toll ISCW00727 expression was downregulated by *A. phagocytophilum* (54). The expression of another component of the Toll pathway, MyD88, was also downregulated by infection with these three pathogens, suggesting that they might suppress this pathway to promote vector colonization. To confirm this hypothesis, it is necessary to functionally characterize the role played by Toll components in pathogen proliferation.

#### The Unconventional Immune Deficiency Pathway

In *Drosophila*, bacterial infections caused by Gram-negative bacteria and certain Gram-positive bacteria, such as *Bacillus* and *Listeria* species, are mainly controlled by the IMD pathway through the recognition of diaminopimelic acid (DAP)-type PGN, which is present in the bacterial cell wall, by PGN-recognition proteins (PGRPs) (44, 55). Genomic and *in silico* studies have shown that ticks lack orthologs of many key elements of the IMD pathway, including the transmembrane PGRP, the Fas-associated protein with death domain (FADD), the adaptor molecule IMD, and the death-related ced-3/Nedd2-like protein (DREDD) (31, 33, 38, 48) (**Figure 2B**). Losses of IMD pathway components are not exclusive to ticks since they have also been described in other arachnids and hemipterans (30, 56). Nevertheless, it is important to highlight that some arthropods have unusual gene architectures, resulting in inaccurate annotation due to the use of software based on standard gene structures, as reported for the kissing bug *Rhodnius prolixus* (57). Gathering data from the genome and transcriptome associated with reciprocal BLAST (Basic Local Alignment Search Tool) and hidden Markov model profile searches, the authors showed that most of the missing IMD pathway components are present in this hemipteran. Therefore, it is possible that the missing IMD pathway components might be a consequence of incorrect annotations due to structural divergences. Indeed, assays showed that the IMD cascade is functional in the insect fat body and is predominantly responsive against Gram-negative bacterial infection (57).

Despite missing several elements, the tick IMD pathway is functional and responsive to distinct pathogens (48, 49, 58). RNAi silencing of several IMD pathway components, including Bendless, ubiquitin E2 variant 1A (UEV1a), Relish, and Caspar (**Figure 2B**, insert), showed that this cascade controls *A. phagocytophilum* and *B. burgdorferi* burden in *I. scapularis* nymphs (48). In contrast to the classical *Drosophila* model of DAP-PGN recognition by PGRPs (44, 55), glycerophospholipids from bacterial membranes, including 1-palmitoyl-2-oleoyl-sn-glycerol-3-phosphoglycerol (POPG) and 1-palmitoyl-2-oleoyl diacylglycerol (PODAG), were reported to act as PAMPs for IMD pathway activation in ticks (48) (**Figure 2B**, insert). However, the mechanisms of POPG and PODAG recognition remain unclear, but it is hypothesized that they are sensed by a yet uncharacterized pattern-recognition receptor. X-linked inhibitor of apoptosis protein (XIAP) is an upstream signaling component of the IMD pathway and, when activated, specifically and directly interacts with the heterodimer E2 conjugating enzyme complex Bendless : UEV1a (48). Upon microbial activation, XIAP, together with Bendless : UEV1a, binds and ubiquitylates its p47 substrate in a K63-dependent manner. Ubiquitylated p47 connects to Kenny (also named NEMO) and induces, by a yet unknown mechanism, phosphorylation of the inhibitor of NF-κB kinase (IKK) β (also known as IRD5) and Relish, the IMD transcription factor. Consequently, Relish is cleaved and translocated to the nucleus (58) (**Figure 2B**, insert). On the other hand, RNAi knockdown of two other components of the IMD pathway, transforming growth factor-β activated kinase 1 (TAK1) and TAK1 adaptor protein 1 (TAB1) (**Figure 2B**), presented no effect on the *A. phagocytophilum* burden in the salivary glands of *I. scapularis* nymphs (53). Therefore, studies carried out by Dr. Pedra’s group (48, 58) showed how the IMD pathway is activated in ticks, which is highly relevant since there is a lack of components in this pathway, different from the classic *Drosophila* model (44). However, the effector molecule(s) regulated by the IMD pathway that control(s) infections by pathogens such as *A. phagocytophilum* and *B. burgdorferi* still need to be identified.

In adult *R. microplus*, RNAi silencing of immune signaling of the Toll, IMD, and JAK/STAT pathway transcription factors identified the IMD pathway as the main controller of *A. marginale* infection in the tick gut and salivary glands (49). The expression of the genes encoding the AMPs microplusin, defensin, ixodidin, and lysozyme was analyzed in the gut and salivary glands of *R. microplus* after knockdown of Relish and infection with *A. marginale*. Interestingly, only the microplusin transcript levels were downregulated in dsRelish ticks, implicating this AMP as an effector of the IMD signaling pathway, which may act against *A. marginale* (49). However, although microplusin appears to be under IMD pathway regulation, possible coregulation by the JAK/STAT pathway cannot be discarded (49).

The other branch that constitutes the IMD pathway is JNK signaling (**Figure 2B**). In *Drosophila*, JNK has been shown to be involved in a wide range of biological processes, including cellular immune and stress responses, but it seems to not be required to induce AMP gene expression (59). Although activation of both the JNK and Relish branches of the IMD pathway occurs *via* TAK1 in *Drosophila* (59), additional studies are warranted to determine the activation of JNK pathways in ticks (48, 53, 58).

### JAK/STAT Pathway: Just a Support Molecular Circuit?

In *Drosophila*, the JAK/STAT signaling pathway only plays an indirect role in controlling bacterial and fungal infection. Therefore, this pathway is considered a support circuit to the Toll and IMD pathways; however, it is especially sensitive to viral infections (60). Beyond its effects on the immune response, the JAK/STAT signaling pathway also regulates multiple biological processes, including repair and renewal of the gut epithelial layer (61), a function that was also reported to occur in ticks (62).

Although still poorly understood, the tick JAK/STAT pathway **(****Figure 2C****)** was reported to be functional, playing an important role in the control of pathogens (53, 62, 63). However, it is not clear how ticks activate the JAK/STAT signaling pathway, as the unpaired (Upd) encoding gene, a cytokine-like signaling molecule ligand of the transmembrane receptor Domeless, is missing. In *I. scapularis*, knockdown of the transcription factor STAT and JAK yielded evidence that this pathway is key to the control of *A. phagocytophilum* infection (53). The results also showed that the 5.3-kDa AMP is an effector regulated by the JAK/STAT pathway, which is essential to restrict *A. phagocytophilum* proliferation in tick salivary glands and hemolymph but not in the gut, indicating that additional effectors under JAK/STAT pathway regulation are required in this organ (53). Interestingly, it was reported that *I. scapularis* employs a sophisticated immune strategy that uses a vertebrate host-derived cytokine to stimulate its own JAK/STAT immune pathway (63). During feeding, the interferon-gamma (INFγ) acquired within the infected bloodmeal activates STAT by a yet unknown receptor and, through mediation of a Rho-like GTPase, leads to the synthesis of the AMP domesticated amidase effector 2 (Dae2), limiting the level of *B. burgdorferi*. Other evidence that indicates that the JAK/STAT pathway is associated with the regulation of AMPs was reported by Capelli-Peixoto and collaborators in adult *R. microplus* (49). The authors observed the downregulation of the AMPs ixodidin and lysozyme in the salivary glands and defensin in the gut and salivary glands of STAT-deficient ticks.

Effectors from signaling pathways, such as JAK/STAT, can act as either positive or negative regulators of infection. As presented above, Dae2 (63) and the 5.3-kDa AMP (53) are negative regulators, as they control pathogen proliferation. In contrast, peritrophin-1, another effector from the tick JAK/STAT pathway, was reported to increase *B. burgdorferi* survival in the gut of *I. scapularis* nymphs (62). Knockdown of STAT had a direct impact on the gut epithelium, affecting its mitotic activity as well as decreasing peritrophin-1 expression, which consequently disrupted the structural integrity of the peritrophic matrix (62). Therefore, peritrophin-1, which is a component of the peritrophic matrix, favors *B. burgdorferi* establishment (62). Interestingly, peritrophin-1 exhibits the opposite effect on *A. phagocytophilum*, another pathogen naturally transmitted by *I. scapularis* (64). Infection with *A. phagocytophilum* upregulates a tick antifreeze glycoprotein, which, in turn, alters bacterial biofilm formation and, consequently, disturbs the natural gut microbiota. This microbiota alteration affects the integrity of the peritrophic matrix, favoring pathogen colonization (64). Knockdown of peritrophin-1 and, therefore, the reduction in the thickness of the peritrophic matrix increases the *A. phagocytophilum* load in the tick gut (64).

### RNAi as a Tick Innate Immunity Component

RNAi is a biological process that plays an important role in the defense of arthropods against viruses and transposable elements. Four main RNAi-related pathways have been described based on the origin of the activating small RNAs. The origin of three of these small RNAs is endogenous [microRNA (miRNA), small interfering RNA (endo-siRNA), and piwi-interacting RNA (piRNA)], while the origin of the fourth is exogenous (siRNA) (65). The exogenous siRNA pathway is especially important and has been proposed to be the main antiviral response in *Drosophila* and mosquitoes (66). In general, after infection, long viral dsRNA is recognized and cleaved by Dicer-2 (Dcr-2) into 21 nucleotide (nt) siRNAs, known as viRNAs (65, 66). These viRNAs are then transferred to Argonaute-2 (Ago2), which couples to other members of the RNA-induced silencing complex (RISC). Only one strand of the viRNA remains coupled to RISC and guides the degradation of complementary viral RNA (65, 66). miRNAs use a similar mechanism, although involving Dcr-1 and Ago-1 (67).

The genome of *I. scapularis* exhibits significant gene expansion in RNAi elements, including five *Ago* homologous genes: *Ago-78*, homologous to insect *Ago-1*, and *Ago-96*, *-68*, *-16*, and*-30*, homologous to insect *Ago-2* (68). Additionally, two *Dcr* genes, *Dcr-89* and *-90*, were clustered with *Drosophila Dcr-2* and *-1*, respectively. Similar gene expansion was identified in *Hyalomma asiaticum* RNAi components (viz., two copies of *Dcr-2* and five copies of *Ago-2*) (69). Infection of *I. scapularis* IDE8 cells with Langat virus (LGTV) showed that Ago-16 and Ago-30 neutralized both LGTV and its replicon, as well as Dcr-90, despite the clustering of the last element with insect Dcr-1, which is involved in miRNA processing but not in siRNA (68). Shortly thereafter, knockdown of Ago-30 and Dcr-90 confirmed their antiviral role upon LGTV infection in *I. scapularis* IDE8 and *I. ricinus* IRE/CTVM19 cell lines (70).

Interestingly, viral or endogenous siRNAs were shown to be mostly 22 nt in length depending on the tick (68), in contrast with *Drosophila* and mosquito viRNAs and endo-siRNAs, which contain 21 nt (66). Moreover, these viRNAs mapped at the highest frequency around the 5’ and 3’ UTRs of the viral genome and antigenome (68). The 3’ UTRs of LGTV and TBEV express subgenomic flavivirus RNAs (sfRNAs), which are a counterdefense against the tick RNAi system, assuring vectorial competence (68). Of note, sfRNAs are expressed by almost all Flaviviridae members as an evolved balance between arthropods and viruses (67).

Grubaugh and collaborators (71) validated the *in vitro* data previously obtained by Schnettler et al. (68), showing that most viRNAs are, indeed, 22 nt in length and originate from the UTR of the viral genome and antigenome of *I. scapularis* in its life stages (larvae, nymphs, and adults) naturally infected with Powassan virus (POWV) (71). Moreover, the viral genetic diversity in ticks is lower than that in mice, suggesting that ticks exert stronger viral control than their vertebrate hosts. Therefore, POWV evolution seems to depend on RNAi-mediated diversification and selective constraints (71).

Regarding endogenous miRNAs, recent studies have shown that pathogens, such as viruses (72) and bacteria (73), modulate tick miRNA profiles, with a potential role in controlling pathogen replication within the vector (72, 73). On the other hand, the piRNA response to infection is still unknown in ticks. Nonetheless, the piRNA response has been implicated in the response of mosquitoes to viral infections (74, 75). Moreover, Hess and colleagues (76) suggested that the mosquito piRNA response precedes the RNAi-Dcr-2-dependent (siRNA) response during viral infection. In contrast with siRNAs, piRNA activation seems to be mediated by single-stranded RNAs that are Dcr1- and Dcr2-independent and possibly mediated by the endonuclease activity of Piwi proteins, resulting in 24–30 nt small RNAs, as found in *Drosophila*. In addition to antiviral activity, piRNAs seem to have important roles in controlling the activity of transposable elements in the genome and in the development of reproductive tissues (65). Considering the knowledge of the role played by RNAi in the defense of insects against infections, the tick RNAi system represents a wide and still unexplored field awaiting investigation.

### Independent Immune Pathways or Dynamic and Indispensable Crosstalk?

Although the term crosstalk is commonly applied to the arthropod immunity literature, its definition remains conflicting, and in many cases, the mechanism by which it occurs remains unknown. Here, we consider crosstalk to occur when (i) the same effector is regulated by more than one immune signaling pathway (50, 56, 77) and (ii) the components of a specific immune signaling pathway modulate the components of other pathways (49, 51, 52, 78–80).

The regulation of AMP expression by Toll and IMD pathways was initially established in *Drosophila*, as well documented in the historical review by Imler (24). Originally, it was accepted that AMPs were regulated by a specific immune pathway; however, subsequent studies carried out by different research groups showed that this regulation was more complex than initially known, and crosstalk among immune pathways could occur, as described in the examples below. Although AMPs are mostly regulated by either Toll or IMD pathways in *Drosophila*, it has been reported that some AMP-encoding genes can be activated synergistically by both immune pathways (50) (**Figure 3A**). It was shown that the NF-κB transcription factors Dorsal, DIF, and Relish can dimerize as homo- or heterodimers with varying degrees of efficiency. The DIF-Relish heterodimer mediates the crosstalk between the Toll and IMD pathways, resulting in the activation of effectors from both pathways and, consequently, targeting a broader spectrum of infectious microorganisms (50).

Another example of a certain effector being regulated by more than one immune pathway occurs in the hemipteran stinkbug *Plautia stali* (56). As shown by Nishide and collaborators, knockdown of IMD, as well as Toll pathway components, modulates effectors of both pathways. Interruption of both pathways at the same time had a more conspicuous effect on AMP production, strengthening crosstalk (56). The authors proposed an intriguing hypothesis that the redundancy between these two immune signaling pathways may have predisposed them to and facilitated the loss of some IMD-related genes in *P. stali*.

The crosstalk between RNAi and immune signaling pathways has been shown in recent publications (51, 52, 78, 79). In *Culex* mosquitoes, Dcr-2, a central component of the siRNA pathway, recognizes West-Nile virus (WNV) dsRNA and activates a signaling cascade to stimulate Relish *via* tumor necrosis factor (TNF) receptor-associated factor (TRAF) to increase Vago expression **(****Figure 3B****)** (51, 52). Following this transcriptional upregulation, Vago is secreted from infected cells and acts as a vertebrate cytokine functional homolog, binding to a still unknown cellular receptor in surrounding cells and triggering the JAK/STAT pathway. Activation of the JAK/STAT pathway ultimately results in an appropriate antiviral response in uninfected cells, such as upregulation of *vir-1* and other antiviral genes. These studies thereby revealed a paracrine signaling response mediated by a complex network of crosstalk, opening up several intriguing lines of investigation for future studies on arthropod immunity. Other studies have shown crosstalk between RNAi and the Toll pathway in *Ae aegypti* Aag2 cells (78) and *Drosophila* (79). In the first study, the miRNA aae-miR-375 upregulated Cactus, inhibiting the activation of the NF-κB transcription factor and reducing AMP synthesis, consequently enhancing dengue virus (DENV) infection (78). In *Drosophila*, on the other hand, four distinct members of the miR-310 family directly regulate drosomycin expression, a Toll-derived AMP (79). In addition to the connection between RNAi and signaling pathways, the redundancy of distinct miRNAs cotargeting the same transcript highlights the tight regulation imposed by miRNAs on the innate response.

It was also shown that the transcription factors activator protein 1 (AP-1; from the JNK pathway) and STAT neutralize Relish-mediated activation during the innate immune response in *Drosophila*, which is necessary for a proper and balanced immune response. The mechanism for controlling Relish-mediated transcriptional activation is through the formation of a complex composed of AP-1 and STAT with the dorsal switch protein (Dsp1), which recruits a histone deacetylase to prevent Relish transcription (80).

In ticks, to the best of our knowledge, there is only one study reporting putative crosstalk among the immune signaling pathways, which was reported by Capelli-Peixoto and collaborators (49). The authors showed that knockdown of the transcription factors Dorsal, Relish, or STAT downregulates Relish expression (**Figure 3C**), with a consequent increase in the *A. marginale* load in *R. microplus* salivary glands. In contrast, Dorsal-deficient ticks presented no effects on Relish expression in the gut, where, intriguingly, ticks exhibited only modest silencing of Dorsal itself. Relish levels were also diminished in STAT-deficient guts. Only treatment with dsRelish resulted in specific silencing of its target gene in both the gut and salivary glands (**Figure 3C**). Nonetheless, the *A. marginale* burden was higher in the gut of ticks from all groups (dsDorsal, dsRelish, and dsSTAT) than in the control (49). As similarities among *Dorsal*, *Relish*, and *STAT* gene sequences were insignificant, the authors hypothesized that crosstalk of the immune pathways in ticks might occur to enhance the immune response. However, an off-target effect cannot be completely disregarded. Although the regulation of AMPs by the IMD and JAK/STAT pathways has been established, as already described above, it is still necessary to silence AMP-encoding genes to assign their role in *A. marginale* control. Therefore, the tick immune system, as shown in some insects, is also integrated, versatile, and possibly capable of making a network of connections among innate signaling pathways, giving rise to effective antimicrobial responses.

## Antimicrobial Peptides: May the “Source” Be With You!

AMPs are important effectors of the immune systems of both invertebrates and vertebrates, having a broad spectrum of activity against microorganisms (81). In ticks, the main sites of AMP expression are hemocytes, fat body, gut, ovaries, and salivary glands, where they can be modulated in response to either blood feeding or microbial challenge (82). Several reviews of tick AMPs addressing their characterization, as well as their interaction with microorganisms, have been published in the last decade (11, 33, 83, 84).

Interestingly, ticks use host hemoglobin, one of the most abundant proteins within the blood meal, as a source for the production of antimicrobial-derived fragments (85–88). Hemoglobin-derived AMPs, referred to as hemocidins (89), are produced by the proteolytic activity of aspartic and cysteine (catepsin-L like) proteinases from the tick gut (90). Structural studies with the synthetic amidated hemocidin Hb33-61a of *R. microplus* showed that its α-helical C-terminus is responsible for the permeabilization of the microbial membrane (91). However, it is still unknown whether hemocidins act intracellularly or if they are released to the tick gut lumen, where they can fight against microorganisms.

In addition to hemocidins, ticks also produce endogenous (ribosomally synthesized) AMPs (11, 83). Among the several tick AMPs identified to date, microplusins (also known as hebraeins) are among the most well characterized. Microplusin is a cysteine- and histidine-rich AMP that was first isolated from the hemolymph of adult *R. microplus* (92) and *Amblyomma hebraeum* (93). Microplusin was also identified in the ovaries and eggs of *R. microplus* (94), suggesting that in addition to protecting adults, it may also play a role in the protection of embryos before and after oviposition. Microplusin exhibits an α-helical globular domain and chelates metal ions (95). The bacteriostatic activity of microplusin against the Gram-positive bacterium *M. luteus* was reversed by the addition of copper II but not iron II. Indeed, microplusin interferes with the respiration (a copper-dependent process) of both *M. luteus* (95) and the fungus *Cryptococcus neoformans* (96). Microplusin was also reported to affect melanization and capsule formation, which are important virulence factors of *C. neoformans* (96). Interestingly, knockdown of microplusin increased the load of *R. rickettsii* in *Amblyomma aureolatum* (97). On the other hand, this AMP had no effect on either rickettsial transmission or tick fitness. Defensins compose another class of AMPs that have been described in several tick species, displaying activity against different types of microorganisms [for review, see (11, 83)]. For example, defensin-2 of *Dermacentor variabilis* was shown to protect against another bacterium of the genus *Rickettsia*, *R. montanensis*, as its neutralization with antidefensin-2 IgG increased the rickettisal load in the tick gut (98). Defensin-2 causes permeabilization of the bacterial membrane with consequent leakage of cytoplasmic proteins (98).

Dae2 is an AMP of *I. scapularis* that was acquired by horizontal bacterial gene transfer and has become an important effector to control *B. burgdorferi* infection (99), although it does not exhibit direct action on this pathogen (63). Indeed, it was recently shown that Dae2 is physically unable to overcome the outer membrane structure of the outer membrane of Gram-negative bacteria; thus, it does not present lytic activity against *B. burgdorferi*, suggesting the need for other factors, such as membrane-permeabilizing agents (100). As Dae2 is delivered to the vertebrate host bite site *via* saliva and exhibits strong activity against bacteria usually encountered in the host skin, this AMP may protect ticks from the acquisition and proliferation of host skin microbes (100).

Serine proteinase inhibitors have also been reported to play a role in the arthropod immune system. For instance, serine proteinase inhibitors mediate both coagulation and melanization processes of hemolymph and the production of AMPs (101). In addition, serine proteinases may also exert antimicrobial activity, possibly inhibiting proteinases that microorganisms use to colonize host tissues and evade the immune system (102). The first report of a tick serine proteinase inhibitor with antimicrobial properties was the ixodidin of *R. microplus* (103), which presents the key features of trypsin inhibitor-like domain proteins (104). Interestingly, one Kunitz inhibitor was reported to control *R. montanensis* infection in the gut of *D. variabilis* (105). In contrast to defensin, *D. variabilis* Kunitz-type inhibitors present a bacteriostatic effect on *R. montanensis* (106). Therefore, serine proteinase inhibitors are also used by ticks as powerful antimicrobial molecules.

Despite the diverse nature of molecules used by ticks as antimicrobials, little information on their synthesis regulation is available, as discussed above. Therefore, additional studies on the regulation of tick AMPs by immune signaling pathways are required to better understand their role in the control of distinct pathogens.

## Redox Metabolism as an Important Player in the Infection Control Orchestra

In addition to AMPs, triggering of the production of reactive oxygen species (ROS) and reactive nitrogen species (RNS) in response to infection has been described in several arthropods, such as *Drosophila* (107) and mosquitoes (108). ROS have an essential role in infection-related physiological as well as pathophysiological processes, such as signaling, regulation of tissue injury and inflammation, cell survival, proliferation, differentiation, and apoptosis (109, 110).

In ticks, there is still little available information on ROS metabolism and their impact on pathogen control. Nonetheless, it is recognized that hemocytes produce ROS under stimulation. Gram-positive bacteria, zymosan, and phorbol 12-myristate 13-acetate elicit the production of hydrogen peroxide (H_2_O_2_) and superoxide ($O_{2}^{-}$) by hemocytes of *R. microplus* (111). In contrast, stimulation with lipopolysaccharide (LPS), the major component of the Gram-negative bacterial outer membrane, failed to induce ROS generation, indicating that different mechanisms or roles for ROS upon infection with either Gram-positive or Gram-negative bacteria may exist (111). Further studies with *R. microplus* showed that cytochrome c oxidase subunit III (COXIII), an enzyme of mitochondrial electron transport complex IV involved in mitochondrial ATP and ROS generation, is important for the transmission of *A. marginale* to calves (112). It is possible that COXIII knockdown imbalances tick redox metabolism, affecting its ability to transmit this pathogen (112). The peroxiredoxin Salp25D from *I. scapularis* had no effect on the transmission of *B. burgdorferi* but instead played a role in spirochete acquisition by the tick (113). RNAi-mediated silencing of Salp25D affects bacterial acquisition by ticks fed on *B. burgdorferi*-infected mice. The same effect was obtained when ticks were fed on Salp25-immunized mice (113). It is possible that Salp25 may detoxify ROS at the tick feeding site and gut, thus affording a survival advantage to *B. burgdorferi*.

In the mosquito *An. gambiae*, an extracellular matrix crosslinked by dityrosine covalent bonds catalyzed by dual oxidase (DUOX) and heme peroxidase is located in the gut ectoperitrophic space (between the epithelial cell layer and the peritrophic matrix). This extracellular matrix acts as an additional physical barrier to decrease gut permeability to bacterial PAMPs, impairing immune response activation by the resident microbiota (114). Importantly, the dityrosine network also provides a favorable environment for *Plasmodium* development, as it prevents the activation of nitric oxidase synthase (iNOS), a nitric oxide-generator enzyme (114). iNOS is responsible for parasite nitration, a key step in the action of the antiplasmodium complement-like molecule TEP1. Later, it was shown that the heme peroxidase 2/NADPH oxidase 5 system plays a central role in epithelium nitration, therefore potentiating the antiparasitic effect of nitric oxide (115). Similar to *An. gambiae* (114), an extracellular matrix was described in the tick *I. scapularis*, which acts as a shield that favors *B. burgdorferi* survival and indirectly prevents the induction of borreliacidal agents in the tick gut (116).

Intriguingly, *A. marginale* upregulated the genes encoding antioxidant enzymes, including superoxide dismutase, catalase, glutathione peroxidase, glutathione S-transferase, thioredoxin, thioredoxin reductase, and peroxiredoxin, whereas genes encoding ROS-generating enzymes, such as DUOX and endoplasmic reticulum oxidase, were downregulated in *R. microplus*-derived BME26 cells (117). Conversely, *R. rickettsii* and heat-killed *S. cerevisiae, E. cloacae* or *M. luteus* triggered the opposite gene expression pattern (117). Furthermore, simultaneous RNAi knockdown of catalase, thioredoxin, and glutathione peroxidase, three representative members of the tick antioxidant enzymatic system, as well as the oxidation resistance 1 (OXR1), which regulates the expression of ROS detoxification enzymes, decreased *A. marginale* infection (117). Therefore, while BME26 cells respond to infection, producing an oxidant environment, *A*. *marginale* seems to subvert this response to create an antioxidant environment, which is required for its survival (117). It is possible that *A. marginale* manipulates *R. microplus* redox metabolism (and production of immune signaling pathway effectors, as aforementioned) to favor its proliferation. Additional studies are required to elucidate the mechanisms that this bacterium uses to subvert tick immune responses.

## Cell-Mediated Immunity in Ticks

Hemocytes, which are sessile or circulating cells from arthropod hemolymph, are responsible for several immune responses. The nomenclature of hemocytes varies considerably depending on the arthropod species and/or the approaches of the study (118). Earlier morphological, ultrastructural, and physiological studies of the hemocyte repertoire in different tick species consistently reported the presence of three basic types of hemocytes, namely, phagocytic plasmatocytes and granulocytes and nonphagocytic granulocytes (119–121). These cells apparently differentiate from rarely occurring prohemocytes (120, 122). More recent studies have described additional types of tick hemocytes, namely, adipohemocytes in *Rhipicephalus sanguineus* (123) and spherulocytes and oenocytoids in *R. microplus* (122). The most important immune responses of arthropod hemocytes are phagocytosis, encapsulation, nodulation (which involves melanization by the PPO cascade), coagulation, and production of immune-related molecules.

The role of tick hemocytes in the phagocytosis of a variety of microbes, including bacteria, yeast, spirochetes, and foreign particles, has been investigated by several studies [for example, see (111, 124–127)]. By contrast, very little is known about the encapsulation and nodulation mechanisms. Indeed, there is only one report on encapsulation (128) and one on nodulation (129), both in *D. variabilis*. After the inoculation of ticks with *Escherichia coli*, hemocytes did not form circular layers but aggregated around the bacteria, which is a characteristic feature of nodule formation (129). As the encapsulation study was performed using an implant of Epon−Araldite under the tick cuticle, it is still unknown whether it also occurs against microorganisms (128).

In invertebrates such as insects and crustaceans, hemocytes produce components of the melanization response, which involves an enzymatic cascade referred to as the PPO activating system, ultimately resulting in the production of melanin (130, 131). This process can be locally activated by cuticle injury or systemically triggered by microbial invasion of the hemocoel. Interestingly, in the cotton bollworm *Helicoverpa armigera*, infection with the baculovirus HearNPV decreased the levels of the majority of PPO cascade components, while serpin-9 and serpin-5 (which were also shown to regulate the proteases cSP4 and cSP6, respectively) were increased (132). In addition, *in vitro* assays showed that hemolymph melanization can kill baculovirus, an effect abolished by the specific PO inhibitor phenylthiourea. Together, the results suggest that baculovirus inhibits the melanization response to ensure its survival in *H. armigera* (132). There is no evidence of the existence of the PPO cascade in ticks based on available genomic and transcriptomic data. In line with this, no PPO activity has been reported to be present in the hemolymph of the hard ticks *Amblyomma americanum, D. variabilis*, and *I. scapularis* (133). In contrast, two studies reported PPO-like activity using L-DOPA as a substrate in the hard tick *R. sanguineus* (134) and in the soft tick *Ornithodoros moubata* (135). However, the enzymes responsible for such activity have not yet been identified, and enzymatic assays did not employ phenylthiourea as a control.

Coagulation is another important immune response of arthropods. The final product of coagulation is a protein clot, which is essential to avoid the loss of hemolymph in cases of an injury and the spread of an invader microorganism throughout the hemocoel (136). In horseshoe crabs, the clotting process involves a serine-protease cascade that leads to the activation of the clotting enzyme that converts the coagulogen into the insoluble clot (137), while in crayfish, the process depends on direct transglutaminase (TG)-mediated cross-linking of a specific plasma protein homologous to vitellogenins (19, 136). TG is also involved in the final step of coagulation in horseshoe crabs, cross-linking coagulin with hemocyte surface proteins named proxins (138). Interestingly, factors of the coagulation cascade interact with hemocyanin, causing it to present PO activity in the horseshoe crab *Tachypleus tridentatus*, demonstrating crosstalk between melanization and coagulation cascades (139). In *Drosophila*, coagulation and PO activity were also described to be tightly associated (140). Wound sealing in flies involves two steps: in the first step, TG-mediated crosslinking of hemolymph proteins occurs, and in the second step, PO-dependent crosslinking takes place, hardening the clot and producing melanin. In ticks, putative coagulation was uniquely reported for *D. variabilis*, where a fibrous matrix was observed around an inert implant (128). TGs and proclotting enzyme precursors have been detected in tick genomes (33). Moreover, an injury-responsive multidomain serine protease homologous to *Limulus* Factor C has been characterized in *I. ricinus* (141). Therefore, additional studies based on appropriate *in vitro* assays are needed to ultimately resolve the question of the existence of hemolymph clotting in ticks.

Tick hemocytes (83), as well as the hemocytes of other arthropods, such as mosquitoes (142), also produce a series of immune-related molecules. Intriguingly, the hemocytome of *I. ricinus* showed that only 1.48% of the 15,716 coding sequences (CDSs) identified were related to immune factors (143). Of the identified CDSs, 327 were five times more highly expressed in hemocytes than in salivary glands and the gut, among which 11 encode immune factors, including AMPs and proteins involved in pathogen recognition. As presented in this section, hemocytes are versatile components of the arthropod immune system that play diverse and key roles. The principal insect tissue that produces the majority of soluble immune molecules in hemolymph is the fat body (44). The role of tick fat body in the tick immune system requires further investigation.

## The Primordial Complement System of Ticks

One important branch of both cellular and humoral innate immunity in vertebrate and invertebrate metazoan organisms is carried out by the complement system. In higher vertebrates, the complement system is composed of approximately thirty components arranged in classical, lectin, and alternative pathways, which recognize foreign cells (microbes), specifically tag them *via* opsonization, and ultimately, eliminate them *via* phagocytosis or cell lysis (144). The common denominator of all three pathways is the proteolytic activation of the central C3 complement component. The occurrence of this molecule can be traced back in most ancient invertebrates, such as horseshoe crabs (subphylum Chelicerata, class Merostomata), implying that an ancestor of the complement system existed on Earth for more than 500 mil. years (145, 146). For ticks, which are also chelicerates, advanced knowledge of the primitive complement system of horseshoe crabs gathered during the past two decades presents the best matching comparative model (137, 145, 147).

**Figure 4:**
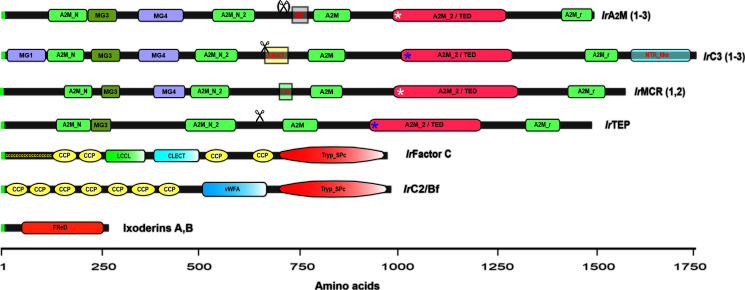
Schematic representation of tick complement-related molecules (TEPs, convertases, lectins). Invertebrate TEPs are divided into four groups: panprotease inhibitors of the α_2_-macroglobulin type (α_2_M); C3-like complement components (C3); macroglobulin complement-related (MCR); and insect-type TEPs (iTEPs). IrA2M(1–3) represents *I. ricinus* α_2_M: IrA2M-1, 2 and 3; IrC3(1-3) represents *I. ricinus* C3: IrC3-1, IrC3-2, and IrC3-3; *Ir*MCR (1,2) represents *I. ricinus* MCR: IrMcr-1 and IrMCR-*2;* and IrTEP represents *I. ricinus* iTEP. Other components of the *I. ricinus* primitive complement system are two putative convertases: IrFactor C, which shows the domain organization of the *I. ricinus* injury-responsive convertase related to *Limulus* Factor C (141), and IrC2/Bf, which shows the domain organization of the *I. ricinus* convertase related to the complement components C2 and/or Bf (156). Ixoderins A and B show the monomer structure of *Ixodes* sp. lectins related to ficolins (155). Domain abbreviations and nomenclature according to the NCBI conserved domain database (https://www.ncbi.nlm.nih.gov/Structure/cdd/wrpsb.cgi) and symbols used in the figure: MG1, 3, 4 – macroglobulin domains 1, 3, 4; A2M_N – MG2 domain of α_2_-macroglobulins; A2M_N_2 – α_2_-macroglobulin family N-terminal region; Scissors – indicate the posttranslational cleavage site (not present in IrA2M-3); BR – bait region of α_2_-macroglobulins (variable by alternative splicing); ANAT – anaphylatoxin homologous domain (signature domain of C3-complement components); LD – low density lipoprotein class A domain (signature domain of MCRs); A2M_2/TED – thioester containing domain; Blue asterisks – thioester bond present; White asterisks – thioester bond absent in IrA2M-2 and IrMCR-1; A2M_r – α_2_-macroglobulin receptor domain; NTR_like – the signature C-terminal domain of C3,C4, and C5 complement components; ccccccccc – the cysteine-rich N-terminal region of *Limulus* Factor C; CCP – complement control protein module (aka short consensus repeats SCRs or SUSHI repeats); LCCL – LCCL domain; CLECT – C-lectin domain; Tryp_Spc – Trypsin-like serine protease; vWFA – Von Willebrand factor A domain; FReD – Fibrinogen-related domain.

Microbial pattern recognition by the vertebrate lectin pathway is mediated by multimeric mannose-binding lectins (MBLs) or ficolins. The horseshoe crab counterparts of mammalian ficolins are lectins named tachylectin-5 or carcinolectin-5 (148–150). These lectins share a fibrinogen-related protein (FRED) with ficolins but lack the N-terminal collagen-like domain responsible for forming complexes with MBL-associated serine proteases (MASPs) (151), which are absent in arthropods (146). The lectin Dorin M, purified from the plasma of the soft tick *O. moubata* (152), was shown to be a clear ortholog of the horseshoe crab tachylectins-5 (153), and similarly to ficolins and tachylectins, it forms high molecular weight multimers in the native state (152). The search for homologous lectins in *I. ricinus* (154) and in the genome of *I. scapularis* (155) revealed the existence of two phylogenetically distinct families, further referred to as ixoderin A and ixoderin B (**Figure 4**). Ixoderin A is mainly present in plasma and is responsible for the hemagglutination of mouse erythrocytes (155). On the other hand, the salivary gland transcriptomes of *I. ricinus* (157, 158) indicate that ixoderin B represents a highly variable multigene family that is preferentially expressed in the salivary glands and secreted into saliva. The function of these FREPs in tick saliva is still obscure, but we can hypothesize that they may play a role in the recognition of a specific tick host.

The central effector molecules of vertebrate and invertebrate complement systems are proteins belonging to the thioester-containing protein (TEP) family, formerly referred to as proteins of the α_2_-macroglobulin superfamily (144, 159, 160). The TEP designation is given due to the presence of a highly reactive β-cysteinyl-γ-glutamyl thioester (TE) bond within a thioester domain. Invertebrate TEPs are divided into four major phylogenetically distinct groups: (i) panprotease inhibitors of the α_2_-macroglobulin type (α_2_M), (ii) C3-like complement components (C3), (iii) insect-type TEPs (iTEPs), and (iv) macroglobulin complement-related proteins (MCRs) (124, 146, 161). Genome-wide screening of the *I. scapularis* genome (124) and the recently available horseshoe crab genomes (162, 163), together with transcriptome data from a variety of arthropods, reveal that all these major groups of TEPs are present in chelicerates, but C3-like molecules are absent in crustaceans and hexapods, while α_2_Ms were lost in the evolution of some insect lineages, such as *Drosophila* and mosquitoes (146).

Orthologs of nine TEPs present in the *I. scapularis* genome (124) were identified in closely related *I. ricinus* (125), and their full CDSs were recently deposited in GenBank: IrA2M-1 (MT779788); IrA2M-2 (MT779789); IrA2M-3 (MT779790); IrTep (MT779791); IrC3-1 (MT779792); IrC3-2 (MT779793); IrC3-3 (MT779793); IrMcr-1 (MT779795); and IrMcr-2 (MT779796). The domain structure of tick TEP representatives is shown in **Figure 4**. The hallmark domain of α_2_Ms is the presence of the bait region (BR), which is cleaved by the target protease. Several bait region alternative splicing variants were reported in the α_2_M region of the soft tick *O. moubata* (164) as well as in IrA2M-1 of *I. ricinus* (165). IrTEP has a domain architecture quite similar to that of IrA2Ms; however, this molecule is phylogenetically more closely related to insect TEPs (124). Tick C3-like molecules (IrC3-1, IrC3-2, IrC3-3) possess two signature domains, namely, the anaphylatoxin domain and C-terminal NTR complement_C345C domain. The MCRs can be clearly identified based on the presence of the short low-density lipoprotein receptor domain (LD), which occurs in the central part of the molecule (**Figure 4**). Tick TEPs are specifically expressed in tick fat body (IrA2M-1, IrA2M-3, IrC3-1, IrC3-2, IrC3-3), tick hemocytes (IrA2M-2, IrA2M-3), salivary glands (IrC3-2, IrMcr-1), and ovaries (IrTEP) (125).

Other characterized components of the *I. ricinus* primitive complement system are two putative convertases: (i) IrC2/Bf (156), which is related to the vertebrate complement components C2 and/or FactorB (Bf) (144) and homologous to convertases from horseshoe crabs (145, 166), and (ii) IrFC (141), homologous to *Limulus* Factor C, which plays a dual function as the factor that triggers the clotting cascade upon sensing Gram-negative bacterial endotoxins and as an LPS-sensitive convertase of the horseshoe crab C3 complement component (147, 167). Both IrC2/Bf and IrFC are multidomain convertases that share the N-terminal trypsin-like domain and numerous CCP modules (complement control protein, aka sushi domains) (**Figure 4**). While IrC2/Bf is mainly expressed in the tick fat body and its expression is responsive to injection of the yeast *Candida albicans* and a variety of *Borrelia* species (156), IrFC is produced by tick hemocytes, and its expression is responsive to any injury, including injection of sterile phosphate-buffered saline, implicating its role in hemolymph clotting and wound healing (141).

RNAi-based functional studies of *I. ricinus* complement components successively deciphered their nonredundant roles in the phagocytosis of different microbes by tick hemocytes (124, 125, 141, 155, 156, 165). Phagocytosis of Gram-negative bacteria represented by the tick pathogen *Chryseobacterium indologenes* (168) depends mainly on the convertase IrFC, which seems to be linked to the IrC3-3 component. Interestingly, phagocytosis of this bacterium is also clearly mediated by α_2_Ms IrAM2-1 and IrAM2-2 by a yet unknown mechanism that likely involves the interaction of these macromolecular protease inhibitors with the potent metalloprotease secreted by the bacterium (168).

A distinct phagocytic pathway dependent on the convertase IrC2/Bf is responsible for the phagocytosis of the yeast *C. albicans* and spirochete *Borrelia*. Phagocytosis of *C. albicans* is further facilitated by IrC3-1 and IrMcr-2, consistent with the reported role of its related molecule MCR (DmTep6) in the phagocytosis of this yeast by *Drosophila* S2 cells (161). Similar to other Gram-negative bacteria, phagocytosis of *Borrelia* is also mediated by IrC3-3. Ixoderins A and B were found to be involved in the phagocytosis of all the tested microbes, except *Borrelia*. Although *Borrelia afzelii* (the principal Lyme disease-causative agent in Europe) is actively phagocytosed by tick hemocytes; neither RNAi-mediated silencing of any tick complement-related molecules nor the total elimination of phagocytosis by preinjection of latex beads have shown any effect on the transmission of these spirochetes to the host (126). These results indirectly support the recent finding that the transmission of *B. afzelii* from infected *I. ricinus* nymphs to naive mice avoids the tick hemocoel and salivary glands and occurs by a direct gut-to-mouthpart route (169). However, it is possible that the tick complement plays a role in the transmission of other tick-borne pathogens, such as intracellular bacteria, including *Anaplasma* spp. and *Rickettsia* spp., or protozoan parasites, including *Babesia* spp. These objectives await an intensive research focus in the future.

## Regulated Cell Death as an Immune Defense

Regulated cell death (RCD) is widely distributed in nature, occurring in both unicellular and multicellular organisms (170). As extensively stated above, the arthropod innate immune system must coordinate pathogen recognition with effector mechanisms to successfully control infection. Nonetheless, over the past decade, several studies have established RCD processes as important mechanisms for the regulation of the immune response as well as the control of infections (171–173). Autophagy, apoptosis, and necrosis are the main types of RCD that have been described to be related to insect immunity in the last few years (173). In this section, we focus on autophagy and apoptosis and their interconnections with immune signaling pathways.

Autophagy is a highly conserved process in which endogenous material (misfolded proteins and aggregates, damaged organelles, and other macromolecules) or exogenous material (such as invading pathogens) are selectively recognized and sequestered within autophagosomes (double-membrane vesicles) that subsequently fuse with lysosomes, leading to cargo degradation (174). Autophagy is executed by a series of evolutionarily conserved autophagy-related (ATG) proteins that have orthologs in eukaryotic organisms ranging from yeasts to humans (174). Studies on *Drosophila* have provided excellent insights into the importance of autophagy during microbial infection (175). For instance, infection with the intracellular bacterium *Listeria monocytogenes* induces autophagy in both hemocytes and a hemocyte-derived *Drosophila* cell line (176). Interestingly, the IMD pathway receptor PGRP-LE is involved in bacterial recognition for autophagy activation. In addition, RNAi-mediated silencing of the autophagy genes *atg5* and *atg1* increases the bacterial load within cells, showing that this pathway is important to control infection. Autophagy is also important to control infection by vesicular stomatitis virus (VSV) in *Drosophila* (177, 178). In this process, the viral glycoprotein VSV-G is recognized by Toll-7, activating autophagy *via* a still unknown pathway that is independent of the canonical Toll, IMD, and JAK/STAT pathways (178). The role played by autophagy in the protection of mosquitoes against viruses is somewhat controversial, with reports suggesting both pro- and antiviral effects (179). In ticks, the expression of *atg* genes was upregulated under starvation in *Haemaphysalis longicornis* (180, 181), *I. scapularis* (182), *R. microplus*, and *A. sculptum* (183), correlating with the classical role of autophagy in stress. However, studies correlating tick autophagy with immune responses still need to be performed.

**Figure 5:**
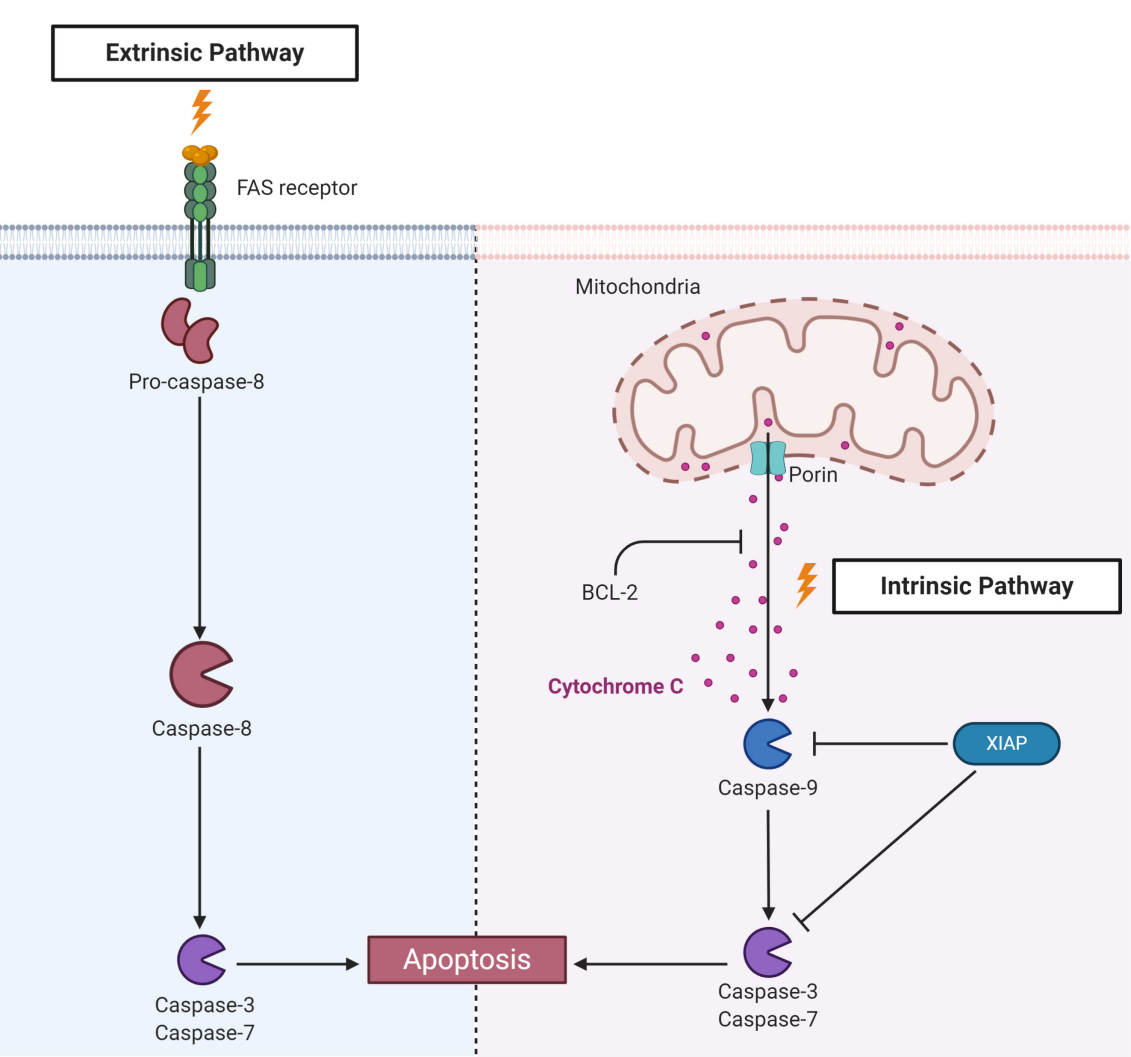
Components of apoptosis activation pathways identified in ticks. Apoptosis is triggered by two main pathways. The extrinsic pathway is activated by recognition of external stimuli by transmembrane death receptors, such as fatty acid synthase (FAS), leading to the activation of caspase-8. The intrinsic pathway, also known as the mitochondrial pathway, is activated by internal stimuli. Subsequently, mitochondrial channels composed, for example, of porins, allow the release of mitochondrial components, such as cytochrome c, to the cytosol, activating the initiator caspase-9. B-cell lymphoma protein 2 (Bcl-2) can inhibit cytochrome c release from mitochondria. Both pathways culminate in the activation of effector or executioner caspases, such as caspases -3 and -7, resulting in chromatin condensation, DNA fragmentation, degradation of nuclear and cytoskeletal proteins and protein cross-linking, which ultimately cause cell death.

Apoptosis is another highly conserved RCD that is essential for removing damaged and infected cells to maintain homeostasis. There are two major apoptotic signaling pathways: extrinsic, also called death receptor pathway, and intrinsic, in which mitochondria play a central role (**Figure 5**). Both of these pathways culminate in the activation of executer caspases that are key for eliminating apoptotic cells (184). Apoptosis is activated in *Drosophila* by infection with *Drosophila* C virus (DCV), and infected cells are phagocytized by hemocytes in a phosphatidylserine-mediated process (185). Apoptosis can also control viral infection in mosquitoes (186, 187). Interestingly, the expression of proapoptotic genes was significantly higher in the refractory strain Cali-MIB of *Ae. aegypti* than in the susceptible strain Cali-S upon experimental infection with DENV-2, suggesting that apoptosis is involved in the distinct susceptibility of mosquitoes to infection (186). Apoptosis is also involved in the control of the proliferation of WNV in the midgut of a refractory strain of the mosquito *Culex pipiens pipiens* (187). Studies on the apoptotic response upon pathogen infection in ticks are also scarcer than those in insects. Infection of the *I. ricinus* cell line IRE/CTVM20 with the bacterium *A. phagocytophilum* and the flaviviruses TBEV and LIV upregulated the expression of apoptosis-associated components, such as cytochrome c and fatty-acid synthase (FAS) (54, 188).

To guarantee their replication and survival within the host cell, many pathogens, including viruses, bacteria and protozoa, subvert apoptosis induced by infection (189). For instance, infection with Zika virus (ZIKV) inhibits apoptosis in *Ae. aegypti* through the action of sfRNAs (190). Until very recently, the unique example of a pathogen that inhibits apoptosis in tick cells was *A. phagocytophilum* (188, 191, 192). This bacterium inhibits the intrinsic apoptosis pathway in *I. scapularis* salivary glands and ISE6 cells by porin (voltage-dependent anion-selective channel) downregulation, resulting in the inhibition of cytochrome c release. Nonetheless, while the intrinsic pathway is inhibited, the extrinsic pathway seems to be activated through the inhibition of FAS by an unknown mechanism as a possible attempt to limit bacterial infection (192, 193). Conversely, in the *I. scapularis* gut and *I. ricinus* IRE/CTVM20 cells, *A. phagocytophilum* supposedly inhibits apoptosis through upregulation of the JAK/STAT pathway (191). However, these conclusions were mostly based on transcriptomics and proteomics data, and only a few genes were functionally characterized by RNAi. In addition, the effectors that *A. phagocytophilum* uses to inhibit tick apoptosis have not been elucidated to date, as they have been for the manipulation of apoptosis in human neutrophils (194). Recently, it was reported that *R. rickettsii* downregulates negative regulators of apoptosis in the initial phase of BME26 cell infection, which are upregulated later. Infection also prevents the fragmentation of DNA and decreases the activity of caspase-3 as well as the exposure of phosphatidylserine. Remarkably, bacterial growth is higher in apoptosis-inhibited tick cells, suggesting that such an inhibitory effect is important to guarantee cell colonization (195).

Apoptosis is closely regulated by apoptosis inhibitor proteins (IAPs) (**Figure 5**) (196, 197). IAPs present at least two conserved motifs: baculoviral IAP repeat (BIR) motifs, which are represented from one to three tandem repeats in the N-terminus, and the C-terminal really interesting new gene (RING) motif; this last motif presents E3-ubiquitin ligase activity. In *Drosophila*, the E3-ubiquitin ligase activity of DIAP-2 has been described as being important for the activation of Relish after recognition of Gram-negative bacteria (198–201). Knockdown of the XIAP of *I. scapularis* from the IMD pathway, which also possesses E3-ubiquitin ligase activity, increased colonization by *A. phagocytophilum*, showing that E3 is important for the control of infection (202) (see the above section “The unconventional IMD pathway”). However, it is still unknown whether XIAP plays a role in tick apoptosis.

Additional studies are warranted to better understand the role played by tick apoptosis pathway components in infection control and their interconnections with immune signaling pathways as well as the mechanisms that pathogens use to subvert the death of tick cells, thereby guaranteeing their survival and proliferation.

## The Role of Tick Microbiota in Vector Competence

Ticks, as well as most multicellular eukaryotes, possess associated bacteria, viruses, fungi and archaea, mainly in mucosal organs, composing their microbiota (203). In the last decade, several studies have focused on the bacterial composition of different genera of ticks and have explored the interaction of TBPs with nonpathogenic tick endosymbionts to elucidate the impact of microbiota on their vector competence (62, 64, 204–206). The tick immune responses to microbiota, despite its importance for a more comprehensive understanding of tick biology, is a field that requires attention since little is known in comparison with other arthropods. Therefore, in this section, we summarize the *Ae. aegypti* immune responses to the gut microbiota and relate this knowledge to ticks.

In adult mosquitoes, the IMD pathway is activated in response to microbiota proliferation induced by the blood meal, limiting Sindbis infection (207). ROS production is mediated by DUOX, whose expression is regulated by a gut membrane-associated protein named Mesh (208). However, a reduction in ROS due to heme release upon blood digestion protects the gut microbiota (209). To counteract the action of Relish-dependent AMPs, the gut microbiota stimulates the expression of C-type lectins (CLTs) in *Ae. aegypti*, which bind to bacterial cell walls, thereby protecting the bacteria (210).

In addition to the immune response to microbiota, there is interest in the impact of microbiota on the vector capacity of mosquitoes and ticks. In *Ae. aegypti*, several studies have shown that larval microbiota can influence vector competence in adult mosquitoes, playing a critical role in their response to viral infections (211). For instance, *E. coli* infection during the larval stage stimulates the production of AMPs and nitric oxide, protecting the mosquito from other infections (212). In addition, when Enterobacteriaceae bacteria are the only members of the larval microbiota, DENV infection in adults is reduced in comparison to *Salmonella* sp. as the only member (213). Conversely, exposure to pathogenic *Bacillus thuringiensis* subsp. *israelensis* in resistant larvae increases adult susceptibility to DENV [but not to Chikungunya (CHIKV)] (214), possibly due to changes in the microbial community (215).

Of all the bacteria present in insect microbiota, *Wolbachia pipientis* may be the most ubiquitous symbiont, as it is naturally present in 40% of all terrestrial arthropod species (216). Intracellular and maternally transmitted *Wolbachia* can cause pathogen interference (PI; the ability to reduce the chance of pathogen infection and decrease pathogen load) and cytoplasmic incompatibility (CI; when infected males mate with uninfected females, the hatch of eggs is heavily reduced), manipulating host reproduction and working as a genetic driver (the ability to spread through a population in a non-Mendelian way) (217). In *Ae. aegypti*, *Wolbachia* strongly reduces CHIKV, DENV and ZIKV infection and vector competence *via* the PI phenotype (217–220). For this reason, there is an ongoing program, the World Mosquito Program, to infect mosquito eggs with *Wolbachia* (from *Drosophila* – *w*Mel) in the laboratory and release them in dengue-endemic areas, such as the city of Rio de Janeiro in Brazil (221, 222).

Despite being the most common bacteria in the microbiota of insects, *Wolbachia* has been reported only in a few species of ticks (205, 223, 224). In fact, the adult tick microbiota is mostly composed of *Coxiella*, *Rickettsia*, *Francisella*, *Spiroplasma*, *Midichloria* and *Rickettsiella* (203, 205). Most of these bacteria are intracellular and noncultivable in the laboratory, which hampers the manipulation of tick microbiota. Some studies on the larval microbiota of *I. scapularis* (62, 64, 204) showed a wide variety of bacterial genera, including cultivable extracellular bacteria. A deep bioinformatics analysis of the raw data of these studies suggested a taxonomic core composed of 61 bacterial taxa for *I. scapularis* larvae (225). However, this high number of bacterial genera in the larval microbiota of *I. scapularis* has been questioned, and the possibility of contamination potentially due to the low biomass of tick samples has been raised (226, 227). Interestingly, some tick species, such as *R. microplus* and *I. ricinus*, present a poor and unstable microbiota in the gut (228). On the other hand, these two species harbor a more abundant and stable microbiota in their ovaries that is composed mostly of *Midichloria* spp. in *I. ricinus* (228) and *Coxiella* spp. in *R. microplus* (229). The authors hypothesized that the reduced microbiota in the tick gut might be due to the action of immune factors, such as AMPs and ROS (228).

As described for mosquitoes, the tick microbiota can exert an effect on vector capacity; bacterial infection can modify the microbiota of its host. In *I. scapularis*, perturbation of the normal gut microbiota decreased the expression of STAT, which, in turn, reduced the expression of peritrophin-1. Since the integrity of the peritrophic matrix is essential to *B. burgdorferi* infection, as previously discussed, alteration of the microbiota reduces borrelial colonization (62). In addition, infection with *B. burgdorferi* promotes the expression of the *I. scapularis* gene *pixr*, which encodes a gut secreted protein with functions in tick biology, such as larval molting and inhibition of biofilm formation (preferentially by Gram-positive bacteria), facilitating the colonization of *B. burgdorferi* in ticks (204). These studies suggest a mutual influence or interconnection between the gut microbiota and *B. burgdorferi* in *I. scapularis*. Conversely, *A. phagocytophilum* infection in this same tick species promotes the expression of an antifreeze protein, which perturbs the gut microbiota and reduces the integrity of the peritrophic matrix (64). In contrast to *B. burgdorferi* (62), an extracellular bacterium that benefits from a preserved peritrophic matrix, *A. phagocytophilum*, which is an obligate intracellular bacterium, reduces the thickness of the peritrophic matrix to colonize the tick gut (64). In the tick *Dermacentor andersoni*, a microbiota alteration was induced by feeding on calves treated with oxytetracycline. Although this treatment did not change the microbiota composition, the proportion of its components was altered, negatively impacting the acquisition of *A. marginale* and *Francisella novicida* (230). Importantly, perturbation of the *D. andersoni* microbiota exerted a negative impact on the reproductive fitness of the tick, thereby identifying the microbiota as an important target for the development of control strategies (231).

A recent study compared the microbiota of two *R. rickettsii* tick vectors in Brazil, *Amblyomma sculptum* and *A. aureolatum*, which present significant differences regarding their susceptibility to infection (232). Interestingly, *A. aureolatum* is highly susceptible to *R. rickettsii* infection and harbors a robust intestinal microbiota, mainly composed of the *Francisella* genus. *A. sculptum*, on the other hand, is less susceptible to *R. rickettsii* infection and harbors a reduced intestinal microbiota (206). Additionally, *R. rickettsii* causes a slight reduction in the microbiota load without changing its composition. It has been reported that the transcriptional gut response of these two ticks to *R. rickettsii* infection is also distinct: while the majority of genes of *A. sculptum*, including immune factors, were upregulated by infection, *A. aureolatum* genes were mostly downregulated (233). Together, these data suggest that the *A. aureolatum* gut microbiota somehow desensitizes the immune system and promotes *R. rickettsii* infection. Interestingly, the presence of *Francisella* endosymbionts positively impacted the establishment of *F. novicida* in *D. andersoni*, and the authors hypothesized that these endosymbionts may suppress the tick immune system, favoring *F. novicida* acquisition (230). Additional studies are needed to confirm this hypothesis and to identify the mechanisms by which microbiota delineate tick susceptibility to infection.

Studies focused on the immune response to tick microbiota are necessary to elucidate this important feature, which is involved in many aspects of tick biology, including its vector capacity, representing a question of public health interest. These responses may involve only one immune signaling pathway or crosstalk of the different pathways, as is suggested to occur in *R. microplus* in response to *A. marginale* infection (49). In addition, we can raise a possible role of DUOX in the control of tick microbiota since this enzyme is present and functional in *I. scapularis* (116).

## Conclusions

To date, studies on the interactions between ticks and TBPs have shown that both the IMD and JAK/STAT pathways are key for the control of bacterial infections (*B. burgdorferi*, *A. marginale* and *A. phagocytophilum*), while the Toll and RNAi pathways might be involved in tick defense against viral infections. Studies on the identification of tick immune system-specific effectors are warranted to describe the mechanisms involved in these signaling pathways. In addition, this review has provided several lines of evidence of interconnections between immune signaling pathways, as well as links among several elements from the innate immunity of arthropods, such as the RNAi system, redox metabolism and microbiota. This review also highlights the importance of bearing in mind a widely integrated, versatile, and complex immune system as a response to infection in ticks, far beyond a canonical and linear pathway.

As mentioned above, most of our knowledge on arthropod immunity comes from *Drosophila* as well as other arthropod studies. Nevertheless, the search for a direct correlation between immune signaling and effector specificity in ticks may result in the absence of new and important mechanisms of the tick immune system. For instance, recent works have suggested that ticks express different types of effectors from immune signaling pathway activation, such as peritrophins, which are key structural components of the gut peritrophic matrix. Moreover, it is extremely important to keep in mind that *Drosophila* is not a vector model and, as such, some aspects of the pathogen-vector interaction cannot be fully modeled. Of relevance, the studies on the innate responses of *Drosophila* follow infections by an intrathoracic injection with large loads of artificial pathogens, greatly contrasting those from ticks, which experience natural pathogens, doses, and routes of infection.

Phagocytosis, AMPs, complement-like molecules and ROS production are also considered important factors for protecting ticks from infection. The microbiota can interfere with tick colonization by pathogens as well. Therefore, it is important to identify the microorganisms that compose the microbiota of different organs of ticks and to determine their influence on the tick immune system as well as on tick vector competence. Interestingly, in some insects, the immune system can be primed by nonpathogenic microorganisms, protecting the animal from subsequent infection with a pathogenic microbe (234). Nonetheless, there is only one report on tick immune system priming to date (48). The authors showed that immune priming with POPG and PODAG protects *I. scapularis* against infection by *A. phagocytophilum* and *D. andersoni* against infection by *A. marginale*. In addition, it was shown that the lipid immune-priming effect is abolished only by the silencing of IMD pathway components but not of the Toll or JAK/STAT pathways, excluding an off-target effect (48). Therefore, the tick immune system and its relationship with microorganisms is a wide and unexplored field to be pursued.

In summary, every aspect concerning the tick immune system and its relation with microorganisms - endosymbionts or pathogens - remains far from completely understood. Despite the vast advances made in recent decades, which have helped us to build parts of this puzzle, working with ticks is still a bright and open field full of possibilities. We expect more groups to work with ticks due to their importance to public health and await the discovery of new knowledge in the next few years. In this way, we will all be able to assemble this extraordinary and complex puzzle.

## Author Contributions

All authors searched the literature and wrote the manuscript. SD and AF conceived the work and the final edition. GS, DP, EE, LM, and PK elaborated the figures. All authors contributed to the article and approved the submitted version.

## Funding

This work was supported by funds from the São Paulo Research Foundation (FAPESP 2013/26450-2) and the National Council for Scientific and Technological Development (CNPq) - the National Institutes of Science and Technology Program in Molecular Entomology (INCTEM 573959/2008-0). AF (309733/2018-9) and SD (304382/2017-5) received CNPq research productivity scholarships. GS and DP received a postdoctoral scholarship from FAPESP (2019/07122-0 and 2018/00652-1, respectively) and EE from the Coordination for the Improvement of Higher Education Personnel (CAPES 88887.321638/2019-00). VU and PK were supported by the Czech Science Foundation (20-05736S).

## Conflict of Interest

The authors declare that the research was conducted in the absence of any commercial or financial relationships that could be construed as a potential conflict of interest.

## References

[1] Dantas-Torres F Fernandes Martins T Munoz-Leal S Onofrio VC Barros-Battesti DM. Ticks (Ixodida: Argasidae, Ixodidae) of Brazil: Updated species checklist and taxonomic keys. Ticks Tick Borne Dis (2019) 10:101252. 10.1016/j.ttbdis.2019.06.012 31255534

[2] Dantas-Torres F. Species Concepts: What about ticks? Trends Parasitol (2018) 34:1017–26. 10.1016/j.pt.2018.09.009 30343986

[3] Koch HG Sauer JR. Quantity of blood ingested by four species of hard ticks (Acari:Ixodidae) fed on domestic dogs. Ann Entomol Soc Am (1984) 77:142–6. 10.1093/aesa/77.2.142

[4] Grisi L Leite RC Martins JR Barros AT Andreotti R Cancado PH. Reassessment of the potential economic impact of cattle parasites in Brazil. Rev Bras Parasitol Vet (2014) 23:150–6. 10.1590/S1984-29612014042 25054492

[5] Bowman AS Sauer JR. Tick salivary glands: function, physiology and future. Parasitology (2004) 129(Suppl):S67–81. 10.1017/s0031182004006468 15938505

[6] Kazimirova M Stibraniova I. Tick salivary compounds: their role in modulation of host defences and pathogen transmission. Front Cell Infect Microbiol (2013) 3:43. 10.3389/fcimb.2013.00043 23971008 PMC3747359

[7] Kotal J Langhansova H Lieskovska J Andersen JF Francischetti IM Chavakis T. Modulation of host immunity by tick saliva. J Proteomics (2015) 128:58–68. 10.1016/j.jprot.2015.07.005 26189360 PMC4619117

[8] Simo L Kazimirova M Richardson J Bonnet SI. The essential role of tick salivary glands and saliva in tick feeding and pathogen transmission. Front Cell Infect Microbiol (2017) 7:281. 10.3389/fcimb.2017.00281 28690983 PMC5479950

[9] Dantas-Torres F Chomel BB Otranto D. Ticks and tick-borne diseases: a one health perspective. Trends Parasitol (2012) 28:437–46. 10.1016/j.pt.2012.07.003 22902521

[10] Stanek G Strle F. Lyme borreliosis-from tick bite to diagnosis and treatment. FEMS Microbiol Rev (2018) 42:233–58. 10.1093/femsre/fux047 29893904

[11] Hajdusek O Sima R Ayllon N Jalovecka M Perner J de la Fuente J. Interaction of the tick immune system with transmitted pathogens. Front Cell Infect Microbiol (2013) 3:26. 10.3389/fcimb.2013.00026 23875177 PMC3712896

[12] Brey PT. The contribution of the Pasteur scholl of insect immunity. In: Brey PT Hultmark D, editors. Molecular Mechanisms of Immune Responses in Insects. London: Chapman & Hall (1998). p. 1–39.

[13] Steiner H Hultmark D Engstrom A Bennich H Boman HG. Sequence and specificity of two antibacterial proteins involved in insect immunity. Nature (1981) 292:246–8. 10.1038/292246a0 7019715

[14] Selsted ME Brown DM DeLange RJ Lehrer RI. Primary structures of MCP-1 and MCP-2, natural peptide antibiotics of rabbit lung macrophages. J Biol Chem (1983) 258:14485–9. 6643497

[15] Selsted ME Harwig SS Ganz T Schilling JW Lehrer RI. Primary structures of three human neutrophil defensins. J Clin Invest (1985) 76:1436–9. 10.1172/JCI112121 PMC424095 4056036

[16] Boman HG Hultmark D. Cell-free immunity in insects. Annu Rev Microbiol (1987) 41:103–26. 10.1146/annurev.mi.41.100187.000535 3318666

[17] Ashida M. The prophenoloxidase cascade in insect immunity. Res Immunol (1990) 141:908–10. 10.1016/0923-2494(90)90191-z 2129210

[18] Johansson MW Soderhall K. Cellular immunity in crustaceans and the proPO system. Parasitol Today (1989) 5:171–6. 10.1016/0169-4758(89)90139-7 15463205

[19] Kopacek P Hall M Soderhall K. Characterization of a clotting protein, isolated from plasma of the freshwater crayfish *Pacifastacus leniusculus*. Eur J Biochem (1993) 213:591–7. 10.1111/j.1432-1033.1993.tb17798.x 8097463

[20] Iwanaga S Miyata T Tokunaga F Muta T. Molecular mechanism of hemolymph clotting system in *Limulus*. Thromb Res (1992) 68:1–32. 10.1016/0049-3848(92)90124-s 1448796

[21] Hoffmann JA Kafatos FC Janeway CA Ezekowitz RA. Phylogenetic perspectives in innate immunity. Science (1999) 284:1313–8. 10.1126/science.284.5418.1313 10334979

[22] Hultmark D. *Drosophila* immunity: paths and patterns. Curr Opin Immunol (2003) 15:12–9. 10.1016/s0952-7915(02)00005-5 12495727

[23] Lemaitre B Nicolas E Michaut L Reichhart JM Hoffmann JA. The dorsoventral regulatory gene cassette spätzle/Toll/cactus controls the potent antifungal response in *Drosophila* adults. Cell (1996) 86:973–83. 10.1016/s0092-8674(00)80172-5 8808632

[24] Imler JL. Overview of *Drosophila* immunity: a historical perspective. Dev Comp Immunol (2014) 42:3–15. 10.1016/j.dci.2013.08.018 24012863

[25] Engstrom Y Kadalayil L Sun SC Samakovlis C Hultmark D Faye I. kappa B-like motifs regulate the induction of immune genes in *Drosophila*. J Mol Biol (1993) 232:327–33. 10.1006/jmbi.1993.1392 8345514

[26] Belvin MP Anderson KV. A conserved signaling pathway: the *Drosophila* Toll-Dorsal pathway. Annu Rev Cell Dev Biol (1996) 12:393–416. 10.1146/annurev.cellbio.12.1.393 8970732

[27] Michel K Kafatos FC. Mosquito immunity against *Plasmodium*. Insect Biochem Mol Biol (2005) 35:677–89. 10.1016/j.ibmb.2005.02.009 15894185

[28] Garcia GR Maruyama SR Malardo T Zangirolamo AF Gardinassi LG. The biology of hematophagous arthropods addressed by molecular high-throughput approaches. Austin J Trop Med Hyg (2015) 1:1004–10.

[29] Christophides GK Zdobnov E Barillas-Mury C Birney E Blandin S Blass C. Immunity-related genes and gene families in *Anopheles gambiae*. Science (2002) 298:159–65. 10.1126/science.1077136 12364793

[30] Palmer WJ Jiggins FM. Comparative genomics reveals the origins and diversity of arthropod immune systems. Mol Biol Evol (2015) 32:2111–29. 10.1093/molbev/msv093 PMC4833078 25908671

[31] Rosa RD Capelli-Peixoto J Mesquita RD Kalil SP Pohl PC Braz GR. Exploring the immune signalling pathway-related genes of the cattle tick *Rhipicephalus microplus*: From molecular characterization to transcriptional profile upon microbial challenge. Dev Comp Immunol (2016) 59:1–14. 10.1016/j.dci.2015.12.018 26724380

[32] Zumaya-Estrada FA Martinez-Barnetche J Lavore A Rivera-Pomar R Rodriguez MH. Comparative genomics analysis of triatomines reveals common first line and inducible immunity-related genes and the absence of Imd canonical components among hemimetabolous arthropods. Parasit Vectors (2018) 11:48. 10.1186/s13071-017-2561-2 29357911 PMC5778769

[33] Smith AA Pal U. Immunity-related genes in *Ixodes scapularis*-perspectives from genome information. Front Cell Infect Microbiol (2014) 4:116. 10.3389/fcimb.2014.00116 25202684 PMC4141456

[34] Barnard AC Nijhof AM Fick W Stutzer C Maritz-Olivier C. RNAi in arthropods: insight into the machinery and applications for understanding the pathogen-vector interface. Genes (Basel) (2012) 3:702–41. 10.3390/genes3040702 PMC3899984 24705082

[35] Sun D Guo Z Liu Y Zhang Y. Progress and prospects of CRISPR/Cas systems in insects and other arthropods. Front Physiol (2017) 8:608. 10.3389/fphys.2017.00608 28932198 PMC5592444

[36] Geraci NS Spencer Johnston J Paul Robinson J Wikel SK Hill CA. Variation in genome size of argasid and ixodid ticks. Insect Biochem Mol Biol (2007) 37:399–408. 10.1016/j.ibmb.2006.12.007 17456435

[37] Barrero RA Guerrero FD Black M McCooke J Chapman B Schilkey F. Gene-enriched draft genome of the cattle tick *Rhipicephalus microplus*: assembly by the hybrid Pacific Biosciences/Illumina approach enabled analysis of the highly repetitive genome. Int J Parasitol (2017) 47:569–83. 10.1016/j.ijpara.2017.03.007 28577881

[38] Gulia-Nuss M Nuss AB Meyer JM Sonenshine DE Roe RM Waterhouse RM. Genomic insights into the *Ixodes scapularis* tick vector of Lyme disease. Nat Commun (2016) 7:10507. 10.1038/ncomms10507 26856261 PMC4748124

[39] Jia N Wang J Shi W Du L Sun Y Zhan W. Large-scale comparative analyses of tick genomes elucidate their genetic diversity and vector capacities. Cell (2020) 182:1328–40.e13. 10.1016/j.cell.2020.07.023 32814014

[40] Romano D Stefanini C Canale A Benelli G. Artificial blood feeders for mosquito and ticks-where from, where to? Acta Trop (2018) 183:43–56. 10.1016/j.actatropica.2018.04.009 29625092

[41] Bell-Sakyi L Darby A Baylis M Makepeace BL. The tick cell biobank: a global resource for in vitro research on ticks, other arthropods and the pathogens they transmit. Ticks Tick Borne Dis (2018) 9:1364–71. 10.1016/j.ttbdis.2018.05.015 PMC6052676 29886187

[42] de la Fuente J Estrada-Pena A Venzal JM Kocan KM Sonenshine DE. Overview: ticks as vectors of pathogens that cause disease in humans and animals. Front Biosci (2008) 13:6938–46. 10.2741/3200 18508706

[43] Verhulst NO Boulanger N JS. Impact of skin microbiome on attractiveness to arthropod vectors and pathogen transmission. In: Boulanger N, editor. Skin and Arthropod Vectors. Academic Press (Cambridge, Massachusetts, EUA) (2018). p. 55–81. 10.1016/B978-0-12-811436-0.00003-4

[44] Lemaitre B Hoffmann J. The host defense of *Drosophila melanogaster*. Annu Rev Immunol (2007) 25:697–743. 10.1146/annurev.immunol.25.022106.141615 17201680

[45] Kitsou C Pal U. *Ixodes* immune responses against Lyme disease pathogens. Front Cell Infect Microbiol (2018) 8:176. 10.3389/fcimb.2018.00176 29896452 PMC5986905

[46] Lindsay SA Wasserman SA. Conventional and non-conventional *Drosophila* Toll signaling. Dev Comp Immunol (2014) 42:16–24. 10.1016/j.dci.2013.04.011 23632253 PMC3787077

[47] Oliva Chavez AS Shaw DK Munderloh UG Pedra JH. Tick humoral responses: marching to the beat of a different drummer. Front Microbiol (2017) 8:223. 10.3389/fmicb.2017.00223 28261180 PMC5306392

[48] Shaw DK Wang X Brown LJ Chavez AS Reif KE Smith AA. Infection-derived lipids elicit an immune deficiency circuit in arthropods. Nat Commun (2017) 8:14401. 10.1038/ncomms14401 28195158 PMC5316886

[49] Capelli-Peixoto J Carvalho DD Johnson WC Scoles GA Fogaca AC Daffre S. The transcription factor Relish controls *Anaplasma marginale* infection in the bovine tick *Rhipicephalus microplus*. Dev Comp Immunol (2017) 74:32–9. 10.1016/j.dci.2017.04.005 28408334

[50] Tanji T Yun EY Ip YT. Heterodimers of NF-kappaB transcription factors DIF and Relish regulate antimicrobial peptide genes in *Drosophila*. Proc Natl Acad Sci USA (2010) 107:14715–20. 10.1073/pnas.1009473107 PMC2930453 20679214

[51] Paradkar PN Duchemin JB Voysey R Walker PJ. Dicer-2-dependent activation of *Culex* Vago occurs via the TRAF-Rel2 signaling pathway. PloS Negl Trop Dis (2014) 8:e2823. 10.1371/journal.pntd.0002823 24762775 PMC3998923

[52] Paradkar PN Trinidad L Voysey R Duchemin JB Walker PJ. Secreted Vago restricts West Nile virus infection in *Culex* mosquito cells by activating the Jak-STAT pathway. Proc Natl Acad Sci USA (2012) 109:18915–20. 10.1073/pnas.1205231109 PMC3503207 23027947

[53] Liu L Dai J Zhao YO Narasimhan S Yang Y Zhang L. *Ixodes scapularis* JAK-STAT pathway regulates tick antimicrobial peptides, thereby controlling the agent of human granulocytic anaplasmosis. J Infect Dis (2012) 206:1233–41. 10.1093/infdis/jis484 PMC3448968 22859824

[54] Mansfield KL Cook C Ellis RJ Bell-Sakyi L Johnson N Alberdi P. Tick-borne pathogens induce differential expression of genes promoting cell survival and host resistance in *Ixodes ricinus* cells. Parasit Vectors (2017) 10:81. 10.1186/s13071-017-2011-1 28202075 PMC5312269

[55] Kleino A Silverman N. The *Drosophila* IMD pathway in the activation of the humoral immune response. Dev Comp Immunol (2014) 42:25–35. 10.1016/j.dci.2013.05.014 23721820 PMC3808521

[56] Nishide Y Kageyama D Yokoi K Jouraku A Tanaka H Futahashi R. Functional crosstalk across IMD and Toll pathways: insight into the evolution of incomplete immune cascades. Proc Biol Sci (2019) 286:20182207. 10.1098/rspb.2018.2207 30963836 PMC6408883

[57] Salcedo-Porras N Guarneri A Oliveira PL Lowenberger C. *Rhodnius prolixus*: identification of missing components of the IMD immune signaling pathway and functional characterization of its role in eliminating bacteria. PloS One (2019) 14:e0214794. 10.1371/journal.pone.0214794 30943246 PMC6447187

[58] McClure Carroll EE Wang X Shaw DK O’Neal AJ Oliva Chavez AS Brown LJ. p47 licenses activation of the immune deficiency pathway in the tick *Ixodes scapularis*. Proc Natl Acad Sci USA (2019) 116:205–10. 10.1073/pnas.1808905116 PMC6320499 30559180

[59] Silverman N Zhou R Erlich RL Hunter M Bernstein E Schneider D. Immune activation of NF-kappaB and JNK requires *Drosophila* TAK1. J Biol Chem (2003) 278:48928–34. 10.1074/jbc.M304802200 14519762

[60] Dostert C Jouanguy E Irving P Troxler L Galiana-Arnoux D Hetru C. The Jak-STAT signaling pathway is required but not sufficient for the antiviral response of *Drosophila*. Nat Immunol (2005) 6:946–53. 10.1038/ni1237 16086017

[61] Osman D Buchon N Chakrabarti S Huang YT Su WC Poidevin M. Autocrine and paracrine unpaired signaling regulate intestinal stem cell maintenance and division. J Cell Sci (2012) 125:5944–9. 10.1242/jcs.113100 23038775

[62] Narasimhan S Rajeevan N Liu L Zhao YO Heisig J Pan J. Gut microbiota of the tick vector *Ixodes scapularis* modulate colonization of the Lyme disease spirochete. Cell Host Microbe (2014) 15:58–71. 10.1016/j.chom.2013.12.001 24439898 PMC3905459

[63] Smith AA Navasa N Yang X Wilder CN Buyuktanir O Marques A. Cross-species interferon signaling boosts microbicidal activity within the tick vector. Cell Host Microbe (2016) 20:91–8. 10.1016/j.chom.2016.06.001 PMC4945435 27374407

[64] Abraham NM Liu L Jutras BL Yadav AK Narasimhan S Gopalakrishnan V. Pathogen-mediated manipulation of arthropod microbiota to promote infection. Proc Natl Acad Sci USA (2017) 114:E781–E90. 10.1073/pnas.1613422114 PMC5293115 28096373

[65] Karlikow M Goic B Saleh MC. RNAi and antiviral defense in *Drosophila*: setting up a systemic immune response. Dev Comp Immunol (2014) 42:85–92. 10.1016/j.dci.2013.05.004 23684730

[66] Blair CD. Mosquito RNAi is the major innate immune pathway controlling arbovirus infection and transmission. Future Microbiol (2011) 6:265–77. 10.2217/fmb.11.11 PMC3126673 21449839

[67] Asgari S. Role of microRNAs in arbovirus/vector interactions. Viruses (2014) 6:3514–34. 10.3390/v6093514 PMC4189037 25251636

[68] Schnettler E Tykalova H Watson M Sharma M Sterken MG Obbard DJ. Induction and suppression of tick cell antiviral RNAi responses by tick-borne flaviviruses. Nucleic Acids Res (2014) 42:9436–46. 10.1093/nar/gku657 PMC4132761 25053841

[69] Yuan C Wu J Peng Y Li Y Shen S Deng F. Transcriptome analysis of the innate immune system of Hyalomma asiaticum. J Invertebr Pathol (2020) 177:107481. 10.1016/j.jip.2020.107481 33035534

[70] Weisheit S Villar M Tykalova H Popara M Loecherbach J Watson M. *Ixodes scapularis* and *Ixodes ricinus* tick cell lines respond to infection with tick-borne encephalitis virus: transcriptomic and proteomic analysis. Parasit Vectors (2015) 8:599. 10.1186/s13071-015-1210-x 26582129 PMC4652421

[71] Grubaugh ND Ruckert C Armstrong PM Bransfield A Anderson JF Ebel GD. Transmission bottlenecks and RNAi collectively influence tick-borne flavivirus evolution. Virus Evol (2016) 2:vew033. 10.1093/ve/vew033 28058113 PMC5210029

[72] Hermance ME Widen SG Wood TG Thangamani S. *Ixodes scapularis* salivary gland microRNAs are differentially expressed during Powassan virus transmission. Sci Rep (2019) 9:13110. 10.1038/s41598-019-49572-5 31511580 PMC6739385

[73] Artigas-Jeronimo S Alberdi P Villar Rayo M Cabezas-Cruz A Prados PJE Mateos-Hernandez L. *Anaplasma phagocytophilum* modifies tick cell microRNA expression and upregulates isc-mir-79 to facilitate infection by targeting the roundabout protein 2 pathway. Sci Rep (2019) 9:9073. 10.1038/s41598-019-45658-2 31235752 PMC6591238

[74] Morazzani EM Wiley MR Murreddu MG Adelman ZN Myles KM. Production of virus-derived ping-pong-dependent piRNA-like small RNAs in the mosquito soma. PloS Pathog (2012) 8:e1002470. 10.1371/journal.ppat.1002470 22241995 PMC3252369

[75] Vodovar N Bronkhorst AW van Cleef KW Miesen P Blanc H van Rij RP. Arbovirus-derived piRNAs exhibit a ping-pong signature in mosquito cells. PloS One (2012) 7:e30861. 10.1371/journal.pone.0030861 22292064 PMC3265520

[76] Hess AM Prasad AN Ptitsyn A Ebel GD Olson KE Barbacioru C. Small RNA profiling of Dengue virus-mosquito interactions implicates the PIWI RNA pathway in anti-viral defense. BMC Microbiol (2011) 11:45. 10.1186/1471-2180-11-45 21356105 PMC3060848

[77] Tanji T Hu X Weber AN Ip YT. Toll and IMD pathways synergistically activate an innate immune response in *Drosophila melanogaster*. Mol Cell Biol (2007) 27:4578–88. 10.1128/MCB.01814-06 PMC1900069 17438142

[78] Hussain M Walker T O’Neill SL Asgari S. Blood meal induced microRNA regulates development and immune associated genes in the Dengue mosquito vector, *Aedes aegypti*. Insect Biochem Mol Biol (2013) 43:146–52. 10.1016/j.ibmb.2012.11.005 23202267

[79] Li Y Li S Li R Xu J Jin P Chen L. Genome-wide miRNA screening reveals miR-310 family members negatively regulate the immune response in *Drosophila melanogaster* via co-targeting drosomycin. Dev Comp Immunol (2017) 68:34–45. 10.1016/j.dci.2016.11.014 27871832

[80] Kim LK Choi UY Cho HS Lee JS Lee WB Kim J. Down-regulation of NF-kappaB target genes by the AP-1 and STAT complex during the innate immune response in *Drosophila*. PloS Biol (2007) 5:e238. 10.1371/journal.pbio.0050238 17803358 PMC1964775

[81] Bulet P Stocklin R Menin L. Anti-microbial peptides: from invertebrates to vertebrates. Immunol Rev (2004) 198:169–84. 10.1111/j.0105-2896.2004.0124.x 15199962

[82] Sonenshine DE Hynes WL. Molecular characterization and related aspects of the innate immune response in ticks. Front Biosci (2008) 13:7046–63. 10.2741/3209 18508715

[83] Kopacek P Hajdusek O Buresova V Daffre S. Tick innate immunity. Adv Exp Med Biol (2010) 708:137–62. 10.1007/978-1-4419-8059-5_8 21528697

[84] Sonenshine DE Macaluso KR. Microbial invasion vs. tick immune regulation. Front Cell Infect Microbiol (2017) 7:390. 10.3389/fcimb.2017.00390 28929088 PMC5591838

[85] Fogaca AC da Silva PI Jr Miranda MT Bianchi AG Miranda A Ribolla PE. Antimicrobial activity of a bovine hemoglobin fragment in the tick *Boophilus microplus*. J Biol Chem (1999) 274:25330–4. 10.1074/jbc.274.36.25330 10464258

[86] Nakajima Y Ogihara K Taylor D Yamakawa M. Antibacterial hemoglobin fragments from the midgut of the soft tick, *Ornithodoros moubata* (Acari: Argasidae). J Med Entomol (2003) 40:78–81. 10.1603/0022-2585-40.1.78 12597657

[87] Sonenshine DE Hynes WL Ceraul SM Mitchell R Benzine T. Host blood proteins and peptides in the midgut of the tick *Dermacentor variabilis* contribute to bacterial control. Exp Appl Acarol (2005) 36:207–23. 10.1007/s10493-005-2564-0 16132735

[88] Belmonte R Cruz CE Pires JR Daffre S. Purification and characterization of Hb 98-114: a novel hemoglobin-derived antimicrobial peptide from the midgut of *Rhipicephalus (Boophilus) microplus*. Peptides (2012) 37:120–7. 10.1016/j.peptides.2012.05.017 22749988

[89] Dubin A Mak P Dubin G Rzychon M Stec-Niemczyk J Wladyka B. New generation of peptide antibiotics. Acta Biochim Pol (2005) 52:633–8. 16175238

[90] Cruz CE Fogaca AC Nakayasu ES Angeli CB Belmonte R Almeida IC. Characterization of proteinases from the midgut of *Rhipicephalus (Boophilus) microplus* involved in the generation of antimicrobial peptides. Parasit Vectors (2010) 3:63. 10.1186/1756-3305-3-63 20663211 PMC2921360

[91] Machado A Sforca ML Miranda A Daffre S Pertinhez TA Spisni A. Truncation of amidated fragment 33-61 of bovine alpha-hemoglobin: effects on the structure and anticandidal activity. Biopolymers (2007) 88:413–26. 10.1002/bip.20688 17245752

[92] Fogaca AC Lorenzini DM Kaku LM Esteves E Bulet P Daffre S. Cysteine-rich antimicrobial peptides of the cattle tick *Boophilus microplus*: isolation, structural characterization and tissue expression profile. Dev Comp Immunol (2004) 28:191–200. 10.1016/j.dci.2003.08.001 14642886

[93] Lai R Takeuchi H Lomas LO Jonczy J Rigden DJ Rees HH. A new type of antimicrobial protein with multiple histidines from the hard tick, *Amblyomma hebraeum*. FASEB J (2004) 18:1447–9. 10.1096/fj.03-1154fje 15247144

[94] Esteves E Fogaca AC Maldonado R Silva FD Manso PP Pelajo-Machado M. Antimicrobial activity in the tick *Rhipicephalus (Boophilus) microplus* eggs: Cellular localization and temporal expression of microplusin during oogenesis and embryogenesis. Dev Comp Immunol (2009) 33:913–9. 10.1016/j.dci.2009.02.009 19454333

[95] Silva FD Rezende CA Rossi DC Esteves E Dyszy FH Schreier S. Structure and mode of action of microplusin, a copper II-chelating antimicrobial peptide from the cattle tick *Rhipicephalus (Boophilus) microplus*. J Biol Chem (2009) 284:34735–46. 10.1074/jbc.M109.016410 PMC2787336 19828445

[96] Silva FD Rossi DC Martinez LR Frases S Fonseca FL Campos CB. Effects of microplusin, a copper-chelating antimicrobial peptide, against *Cryptococcus neoformans*. FEMS Microbiol Lett (2011) 324:64–72. 10.1111/j.1574-6968.2011.02386.x 22092765

[97] Martins LA Malossi CD Galletti M Ribeiro JM Fujita A Esteves E. The transcriptome of the salivary glands of *Amblyomma aureolatum* reveals the antimicrobial peptide microplusin as an important factor for the tick protection against *Rickettsia rickettsii* infection. Front Physiol (2019) 10:529. 10.3389/fphys.2019.00529 31130872 PMC6509419

[98] Pelc RS McClure JC Sears KT Chung A Rahman MS Ceraul SM. Defending the fort: a role for defensin-2 in limiting *Rickettsia montanensis* infection of *Dermacentor variabilis*. Insect Mol Biol (2014) 23:457–65. 10.1111/imb.12094 PMC4106998 24779891

[99] Chou S Daugherty MD Peterson SB Biboy J Yang Y Jutras BL. Transferred interbacterial antagonism genes augment eukaryotic innate immune function. Nature (2015) 518:98–101. 10.1038/nature13965 25470067 PMC4713192

[100] Hayes BM Radkov AD Yarza F Flores S Kim J Zhao Z. Ticks resist skin commensals with immune factor of bacterial origin. Cell (2020) 183:1562–71.e12. 10.1016/j.cell.2020.10.042 33306955 PMC8034492

[101] Gulley MM Zhang X Michel K. The roles of serpins in mosquito immunology and physiology. J Insect Physiol (2013) 59:138–47. 10.1016/j.jinsphys.2012.08.015 PMC3560325 22960307

[102] Armstrong PB. The contribution of proteinase inhibitors to immune defense. Trends Immunol (2001) 22:47–52. 10.1016/s1471-4906(00)01803-2 11286692

[103] Fogaca AC Almeida IC Eberlin MN Tanaka AS Bulet P Daffre S. Ixodidin, a novel antimicrobial peptide from the hemocytes of the cattle tick *Boophilus microplus* with inhibitory activity against serine proteinases. Peptides (2006) 27:667–74. 10.1016/j.peptides.2005.07.013 16191451

[104] Bania J Stachowiak D Polanowski A. Primary structure and properties of the cathepsin G/chymotrypsin inhibitor from the larval hemolymph of *Apis mellifera*. Eur J Biochem (1999) 262:680–7. 10.1046/j.1432-1327.1999.00406.x 10411628

[105] Ceraul SM Chung A Sears KT Popov VL Beier-Sexton M Rahman MS. A Kunitz protease inhibitor from *Dermacentor variabilis*, a vector for spotted fever group rickettsiae, limits *Rickettsia montanensis* invasion. Infect Immun (2011) 79:321–9. 10.1128/IAI.00362-10 PMC3019897 20956566

[106] Ceraul SM Dreher-Lesnick SM Mulenga A Rahman MS Azad AF. Functional characterization and novel rickettsiostatic effects of a Kunitz-type serine protease inhibitor from the tick *Dermacentor variabilis*. Infect Immun (2008) 76:5429–35. 10.1128/IAI.00866-08 PMC2573381 18779339

[107] Ha EM Oh CT Bae YS Lee WJ. A direct role for dual oxidase in *Drosophila* gut immunity. Science (2005) 310:847–50. 10.1126/science.1117311 16272120

[108] Molina-Cruz A DeJong RJ Charles B Gupta L Kumar S Jaramillo-Gutierrez G. Reactive oxygen species modulate *Anopheles gambiae* immunity against bacteria and *Plasmodium*. J Biol Chem (2008) 283:3217–23. 10.1074/jbc.M705873200 18065421

[109] Fang FC. Antimicrobial reactive oxygen and nitrogen species: concepts and controversies. Nat Rev Microbiol (2004) 2:820–32. 10.1038/nrmicro1004 15378046

[110] Jones DP. Redefining oxidative stress. Antioxid Redox Signal (2006) 8:1865–79. 10.1089/ars.2006.8.1865 16987039

[111] Pereira LS Oliveira PL Barja-Fidalgo C Daffre S. Production of reactive oxygen species by hemocytes from the cattle tick *Boophilus microplus*. Exp Parasitol (2001) 99:66–72. 10.1006/expr.2001.4657 11748959

[112] Bifano TD Ueti MW Esteves E Reif KE Braz GR Scoles GA. Knockdown of the *Rhipicephalus microplus* cytochrome c oxidase subunit III gene is associated with a failure of *Anaplasma marginale* transmission. PloS One (2014) 9:e98614. 10.1371/journal.pone.0098614 24878588 PMC4039488

[113] Narasimhan S Sukumaran B Bozdogan U Thomas V Liang X DePonte K. A tick antioxidant facilitates the Lyme disease agent’s successful migration from the mammalian host to the arthropod vector. Cell Host Microbe (2007) 2:7–18. 10.1016/j.chom.2007.06.001 18005713 PMC2699493

[114] Kumar S Molina-Cruz A Gupta L Rodrigues J Barillas-Mury C. A peroxidase/dual oxidase system modulates midgut epithelial immunity in *Anopheles gambiae*. Science (2010) 327:1644–8. 10.1126/science.1184008 PMC3510679 20223948

[115] Oliveira Gde A Lieberman J Barillas-Mury C. Epithelial nitration by a peroxidase/NOX5 system mediates mosquito antiplasmodial immunity. Science (2012) 335:856–9. 10.1126/science.1209678 PMC3444286 22282475

[116] Yang X Smith AA Williams MS Pal U. A dityrosine network mediated by dual oxidase and peroxidase influences the persistence of Lyme disease pathogens within the vector. J Biol Chem (2014) 289:12813–22. 10.1074/jbc.M113.538272 PMC4007469 24662290

[117] Kalil SP Rosa RDD Capelli-Peixoto J Pohl PC Oliveira PL Fogaca AC. Immune-related redox metabolism of embryonic cells of the tick *Rhipicephalus microplus* (BME26) in response to infection with *Anaplasma marginale*. Parasit Vectors (2017) 10:613. 10.1186/s13071-017-2575-9 29258559 PMC5738103

[118] Hillyer JF Christensen BM. Characterization of hemocytes from the Yellow Fever mosquito, *Aedes aegypti*. Histochem Cell Biol (2002) 117:431–40. 10.1007/s00418-002-0408-0 12029490

[119] Kuhn KH Haug T. Ultrastructural, cytochemical, and immunocytochemical characterization of haemocytes of the hard tick *Ixodes ricinus* (Acari; Chelicerata). Cell Tissue Res (1994) 277:493–504. 10.1007/BF00300222

[120] Borovickova B Hypsa V. Ontogeny of tick hemocytes: a comparative analysis of *Ixodes ricinus* and *Ornithodoros moubata*. Exp Appl Acarol (2005) 35:317–33. 10.1007/s10493-004-2209-8 15969464

[121] Inoue N Hanada K Tsuji N Igarashi I Nagasawa H Mikami T. Characterization of phagocytic hemocytes in *Ornithodoros moubata* (Acari: Ixodidae). J Med Entomol (2001) 38:514–9. 10.1603/0022-2585-38.4.514 11476331

[122] Fiorotti J Menna-Barreto RFS Golo PS Coutinho-Rodrigues CJB Bitencourt ROB Spadacci-Morena DD. Ultrastructural and cytotoxic effects of *Metarhizium robertsii* infection on *Rhipicephalus microplus* hemocytes. Front Physiol (2019) 10:654. 10.3389/fphys.2019.00654 31191351 PMC6548823

[123] Feitosa AP Alves LC Chaves MM Veras DL Silva EM Alianca AS. Hemocytes of *Rhipicephalus sanguineus* (Acari: Ixodidae): characterization, population abundance, and ultrastructural changes following challenge with *Leishmania infantum*. J Med Entomol (2015) 52:1193–202. 10.1093/jme/tjv125 26336264

[124] Buresova V Hajdusek O Franta Z Loosova G Grunclova L Levashina EA. Functional genomics of tick thioester-containing proteins reveal the ancient origin of the complement system. J Innate Immun (2011) 3:623–30. 10.1159/000328851 21811049

[125] Urbanova V Sima R Sauman I Hajdusek O Kopacek P. Thioester-containing proteins of the tick *Ixodes ricinus*: gene expression, response to microbial challenge and their role in phagocytosis of the yeast *Candida albicans*. Dev Comp Immunol (2015) 48:55–64. 10.1016/j.dci.2014.09.004 25224405

[126] Urbanova V Hajdusek O Honig Mondekova H Sima R Kopacek P. Tick thioester-containing proteins and phagocytosis do not affect transmission of *Borrelia afzelii* from the competent vector *Ixodes ricinus*. Front Cell Infect Microbiol (2017) 7:73. 10.3389/fcimb.2017.00073 28361038 PMC5352706

[127] Dunham-Ems SM Caimano MJ Pal U Wolgemuth CW Eggers CH Balic A. Live imaging reveals a biphasic mode of dissemination of *Borrelia burgdorferi* within ticks. J Clin Invest (2009) 119:3652–65. 10.1172/JCI39401 PMC2786795 19920352

[128] Eggenberger LR Lamoreaux WJ Coons LB. Hemocytic encapsulation of implants in the tick *Dermacentor variabilis*. Exp Appl Acarol (1990) 9:279–87. 10.1007/BF01193434 2261820

[129] Ceraul SM Sonenshine DE Hynes WL. Resistance of the tick *Dermacentor variabilis* (Acari: Ixodidae) following challenge with the bacterium *Escherichia coli* (Enterobacteriales: Enterobacteriaceae). J Med Entomol (2002) 39:376–83. 10.1603/0022-2585-39.2.376 11931039

[130] Cerenius L Soderhall K. The prophenoloxidase-activating system in invertebrates. Immunol Rev (2004) 198:116–26. 10.1111/j.0105-2896.2004.00116.x 15199959

[131] Nakhleh J El Moussawi L Osta MA. The melanization response in insect immunity. Adv Insect Physiol (2017) 52:83–109. 10.1016/bs.aiip.2016.11.002

[132] Yuan C Xing L Wang M Wang X Yin M Wang Q. Inhibition of melanization by serpin-5 and serpin-9 promotes baculovirus infection in cotton bollworm *Helicoverpa armigera*. PloS Pathog (2017) 13:e1006645. 10.1371/journal.ppat.1006645 28953952 PMC5633200

[133] Zhioua E Browning M Johnson PW Ginsberg HS LeBrun RA. Pathogenicity of the entomopathogenic fungus *Metarhizium anisopliae* (Deuteromycetes) to *Ixodes scapularis* (Acari: Ixodidae). J Parasitol (1997) 83:815–8. 9379283

[134] Feitosa APS Chaves MM Veras DL de Deus DMV Portela NCJ Araujo AR. Assessing the cellular and humoral immune response in *Rhipicephalus sanguineus* sensu lato (Acari: Ixodidae) infected with *Leishmania infantum* (Nicolle, 1908). Ticks Tick Borne Dis (2018) 9:1421–30. 10.1016/j.ttbdis.2018.06.007 30207274

[135] Kadota K Satoh E Ochiai M Inoue N Tsuji N Igarashi I. Existence of phenol oxidase in the argasid tick *Ornithodoros moubata*. Parasitol Res (2002) 88:781–4. 10.1007/s00436-002-0664-x 12122439

[136] Jiravanichpaisal P Lee BL Soderhall K. Cell-mediated immunity in arthropods: hematopoiesis, coagulation, melanization and opsonization. Immunobiology (2006) 211:213–36. 10.1016/j.imbio.2005.10.015 16697916

[137] Iwanaga S Lee BL. Recent advances in the innate immunity of invertebrate animals. J Biochem Mol Biol (2005) 38:128–50. 10.5483/bmbrep.2005.38.2.128 15826490

[138] Osaki T Okino N Tokunaga F Iwanaga S Kawabata S. Proline-rich cell surface antigens of horseshoe crab hemocytes are substrates for protein cross-linking with a clotting protein coagulin. J Biol Chem (2002) 277:40084–90. 10.1074/jbc.M206773200 12189150

[139] Nagai T Kawabata S. A link between blood coagulation and prophenol oxidase activation in arthropod host defense. J Biol Chem (2000) 275:29264–7. 10.1074/jbc.M002556200 10880508

[140] Theopold U Krautz R Dushay MS. The *Drosophila* clotting system and its messages for mammals. Dev Comp Immunol (2014) 42:42–6. 10.1016/j.dci.2013.03.014 23545286

[141] Urbanova V Hartmann D Grunclova L Sima R Flemming T Hajdusek O. IrFC - An *Ixodes ricinus* injury-responsive molecule related to *Limulus* Factor C. Dev Comp Immunol (2014) 46:439–47. 10.1016/j.dci.2014.05.016 24924263

[142] Hillyer JF Strand MR. Mosquito hemocyte-mediated immune responses. Curr Opin Insect Sci (2014) 3:14–21. 10.1016/j.cois.2014.07.002 25309850 PMC4190037

[143] Kotsyfakis M Kopacek P Franta Z Pedra JH Ribeiro JM. Deep sequencing analysis of the *Ixodes ricinus* haemocytome. PloS Negl Trop Dis (2015) 9:e0003754. 10.1371/journal.pntd.0003754 25970599 PMC4430169

[144] Ricklin D Hajishengallis G Yang K Lambris JD. Complement: a key system for immune surveillance and homeostasis. Nat Immunol (2010) 11:785–97. 10.1038/ni.1923 PMC2924908 20720586

[145] Zhu Y Thangamani S Ho B Ding JL. The ancient origin of the complement system. EMBO J (2005) 24:382–94. 10.1038/sj.emboj.7600533 PMC545819 15616573

[146] Sekiguchi R Nonaka M. Evolution of the complement system in protostomes revealed by de novo transcriptome analysis of six species of Arthropoda. Dev Comp Immunol (2015) 50:58–67. 10.1016/j.dci.2014.12.008 25530095

[147] Kawabata S. Immunocompetent molecules and their response network in horseshoe crabs. Adv Exp Med Biol (2010) 708:122–36. 10.1007/978-1-4419-8059-5_7 21528696

[148] Gokudan S Muta T Tsuda R Koori K Kawahara T Seki N. Horseshoe crab acetyl group-recognizing lectins involved in innate immunity are structurally related to fibrinogen. Proc Natl Acad Sci USA (1999) 96:10086–91. 10.1073/pnas.96.18.10086 PMC17846 10468566

[149] Kawabata S Tsuda R. Molecular basis of non-self recognition by the horseshoe crab tachylectins. Biochim Biophys Acta (2002) 1572:414–21. 10.1016/s0304-4165(02)00322-7 12223283

[150] Zhu Y Ng PM Wang L Ho B Ding JL. Diversity in lectins enables immune recognition and differentiation of wide spectrum of pathogens. Int Immunol (2006) 18:1671–80. 10.1093/intimm/dxl101 17035349

[151] Matsushita M. Ficolins: complement-activating lectins involved in innate immunity. J Innate Immun (2010) 2:24–32. 10.1159/000228160 20375620

[152] Kovar V Kopacek P Grubhoffer L. Isolation and characterization of Dorin M, a lectin from plasma of the soft tick *Ornithodoros moubata*. Insect Biochem Mol Biol (2000) 30:195–205. 10.1016/s0965-1748(99)00107-1 10732987

[153] Rego RO Kovar V Kopacek P Weise C Man P Sauman I. The tick plasma lectin, Dorin M, is a fibrinogen-related molecule. Insect Biochem Mol Biol (2006) 36:291–9. 10.1016/j.ibmb.2006.01.008 16551543

[154] Rego RO Hajdusek O Kovar V Kopacek P Grubhoffer L Hypsa V. Molecular cloning and comparative analysis of fibrinogen-related proteins from the soft tick *Ornithodoros moubata* and the hard tick *Ixodes ricinus*. Insect Biochem Mol Biol (2005) 35:991–1004. 10.1016/j.ibmb.2005.04.001 15979000

[155] Honig Mondekova H Sima R Urbanova V Kovar V Rego ROM Grubhoffer L. Characterization of *Ixodes ricinus* fibrinogen-related proteins (ixoderins) discloses their function in the tick innate immunity. Front Cell Infect Microbiol (2017) 7:509. 10.3389/fcimb.2017.00509 29276701 PMC5727070

[156] Urbanova V Hajdusek O Sima R Franta Z Honig-Mondekova H Grunclova L. IrC2/Bf - A yeast and *Borrelia* responsive component of the complement system from the hard tick *Ixodes ricinus*. Dev Comp Immunol (2018) 79:86–94. 10.1016/j.dci.2017.10.012 29061482

[157] Schwarz A von Reumont BM Erhart J Chagas AC Ribeiro JM Kotsyfakis M. De novo *Ixodes ricinus* salivary gland transcriptome analysis using two next-generation sequencing methodologies. FASEB J (2013) 27:4745–56. 10.1096/fj.13-232140 PMC3834774 23964076

[158] Perner J Kropackova S Kopacek P Ribeiro JMC. Sialome diversity of ticks revealed by RNAseq of single tick salivary glands. PloS Negl Trop Dis (2018) 12:e0006410. 10.1371/journal.pntd.0006410 29652888 PMC5919021

[159] Blandin SA Marois E Levashina EA. Antimalarial responses in *Anopheles gambiae*: from a complement-like protein to a complement-like pathway. Cell Host Microbe (2008) 3:364–74. 10.1016/j.chom.2008.05.007 18541213

[160] Shokal U Eleftherianos I. Evolution and function of thioester-containing proteins and the complement system in the innate immune response. Front Immunol (2017) 8:759. 10.3389/fimmu.2017.00759 28706521 PMC5489563

[161] Stroschein-Stevenson SL Foley E O’Farrell PH Johnson AD. Identification of *Drosophila* gene products required for phagocytosis of *Candida albicans*. PloS Biol (2006) 4:e4. 10.1371/journal.pbio.0040004 16336044 PMC1310651

[162] Simpson SD Ramsdell JS Watson Iii WH Chabot CC. The draft genome and transcriptome of the atlantic horseshoe crab, *Limulus polyphemus*. Int J Genomics (2017) 2017:7636513. 10.1155/2017/7636513 28265565 PMC5317147

[163] Zhou Y Liang Y Yan Q Zhang L Chen D Ruan L. The draft genome of horseshoe crab *Tachypleus tridentatus* reveals its evolutionary scenario and well-developed innate immunity. BMC Genomics (2020) 21:137. 10.1186/s12864-020-6488-1 32041526 PMC7011531

[164] Saravanan T Weise C Sojka D Kopacek P. Molecular cloning, structure and bait region splice variants of alpha2-macroglobulin from the soft tick *Ornithodoros moubata*. Insect Biochem Mol Biol (2003) 33:841–51. 10.1016/s0965-1748(03)00083-3 12878230

[165] Buresova V Hajdusek O Franta Z Sojka D Kopacek P. IrAM-An alpha2-macroglobulin from the hard tick *Ixodes ricinus*: characterization and function in phagocytosis of a potential pathogen *Chryseobacterium indologenes*. Dev Comp Immunol (2009) 33:489–98. 10.1016/j.dci.2008.09.011 18948134

[166] Tagawa K Yoshihara T Shibata T Kitazaki K Endo Y Fujita T. Microbe-specific C3b deposition in the horseshoe crab complement system in a C2/factor B-dependent or -independent manner. PloS One (2012) 7:e36783. 10.1371/journal.pone.0036783 22611464 PMC3351276

[167] Le Saux A Ng PM Koh JJ Low DH Leong GE Ho B. The macromolecular assembly of pathogen-recognition receptors is impelled by serine proteases, via their complement control protein modules. J Mol Biol (2008) 377:902–13. 10.1016/j.jmb.2008.01.045 18279891

[168] Buresova V Franta Z Kopacek P. A comparison of *Chryseobacterium indologenes* pathogenicity to the soft tick *Ornithodoros moubata* and hard tick *Ixodes ricinus*. J Invertebr Pathol (2006) 93:96–104. 10.1016/j.jip.2006.05.006 16793056

[169] Pospisilova T Urbanova V Hes O Kopacek P Hajdusek O Sima R. Tracking of *Borrelia afzelii* transmission from infected *Ixodes ricinus* nymphs to mice. Infect Immun (2019) 87:e00896–18. 10.1128/IAI.00896-18 PMC6529662 30910791

[170] Galluzzi L Vitale I Aaronson SA Abrams JM Adam D Agostinis P. Molecular mechanisms of cell death: recommendations of the Nomenclature Committee on Cell Death 2018. Cell Death Differ (2018) 25:486–541. 10.1038/s41418-017-0012-4 29362479 PMC5864239

[171] Ashida H Mimuro H Ogawa M Kobayashi T Sanada T Kim M. Cell death and infection: a double-edged sword for host and pathogen survival. J Cell Biol (2011) 195:931–42. 10.1083/jcb.201108081 PMC3241725 22123830

[172] Steinert S Levashina EA. Intracellular immune responses of dipteran insects. Immunol Rev (2011) 240:129–40. 10.1111/j.1600-065X.2010.00985.x 21349091

[173] Cooper DM Mitchell-Foster K. Death for survival: what do we know about innate immunity and cell death in insects? Invertebr Surv J (2011) 8:162–72. 10.1111/j.1600-065X.2011.01040.x

[174] Kuo CJ Hansen M Troemel E. Autophagy and innate immunity: insights from invertebrate model organisms. Autophagy (2018) 14:233–42. 10.1080/15548627.2017.1389824 PMC5902216 29130360

[175] Moy RH Cherry S. Antimicrobial autophagy: a conserved innate immune response in *Drosophila*. J Innate Immun (2013) 5:444–55. 10.1159/000350326 PMC3799998 23689401

[176] Yano T Mita S Ohmori H Oshima Y Fujimoto Y Ueda R. Autophagic control of *Listeria* through intracellular innate immune recognition in *Drosophila*. Nat Immunol (2008) 9:908–16. 10.1038/ni.1634 PMC2562576 18604211

[177] Shelly S Lukinova N Bambina S Berman A Cherry S. Autophagy is an essential component of *Drosophila* immunity against vesicular stomatitis virus. Immunity (2009) 30:588–98. 10.1016/j.immuni.2009.02.009 PMC2754303 19362021

[178] Nakamoto M Moy RH Xu J Bambina S Yasunaga A Shelly SS. Virus recognition by Toll-7 activates antiviral autophagy in *Drosophila*. Immunity (2012) 36:658–67. 10.1016/j.immuni.2012.03.003 PMC3334418 22464169

[179] Echavarria-Consuegra L Smit JM Reggiori F. Role of autophagy during the replication and pathogenesis of common mosquito-borne flavi- and alphaviruses. Open Biol (2019) 9:190009. 10.1098/rsob.190009 30862253 PMC6451359

[180] Umemiya R Matsuo T Hatta T Sakakibara S Boldbaatar D Fujisaki K. Autophagy-related genes from a tick, *Haemaphysalis longicornis*. Autophagy (2008) 4:79–81. 10.4161/auto.5143 17938584

[181] Kawano S Umemiya-Shirafuji R Boldbaatar D Matsuoka K Tanaka T Fujisaki K. Cloning and characterization of the autophagy-related gene 6 from the hard tick, *Haemaphysalis longicornis*. Parasitol Res (2011) 109:1341–9. 10.1007/s00436-011-2429-x 21537978

[182] Wang XR Kurtti TJ Oliver JD Munderloh UG. The identification of tick autophagy-related genes in *Ixodes scapularis* responding to amino acid starvation. Ticks Tick Borne Dis (2020) 11:101402. 10.1016/j.ttbdis.2020.101402 32035896 PMC7127957

[183] Moura-Martiniano NO Machado-Ferreira E Gazeta GS Soares CAG. Relative transcription of autophagy-related genes in *Amblyomma sculptum* and *Rhipicephalus microplus* ticks. Exp Appl Acarol (2017) 73:401–28. 10.1007/s10493-017-0193-z 29181673

[184] Elmore S. Apoptosis: a review of programmed cell death. Toxicol Pathol (2007) 35:495–516. 10.1080/01926230701320337 17562483 PMC2117903

[185] Nainu F Tanaka Y Shiratsuchi A Nakanishi Y. Protection of insects against viral infection by apoptosis-dependent phagocytosis. J Immunol (2015) 195:5696–706. 10.4049/jimmunol.1500613 26546607

[186] Ocampo CB Caicedo PA Jaramillo G Ursic Bedoya R Baron O Serrato IM. Differential expression of apoptosis related genes in selected strains of *Aedes aegypti* with different susceptibilities to Dengue virus. PloS One (2013) 8:e61187. 10.1371/journal.pone.0061187 23593426 PMC3622604

[187] Vaidyanathan R Scott TW. Apoptosis in mosquito midgut epithelia associated with *West Nile* virus infection. Apoptosis (2006) 11:1643–51. 10.1007/s10495-006-8783-y 16820968

[188] Alberdi P Mansfield KL Manzano-Roman R Cook C Ayllon N Villar M. Tissue-specific signatures in the transcriptional response to *Anaplasma phagocytophilum* Infection of *Ixodes scapularis* and *Ixodes ricinus* tick cell lines. Front Cell Infect Microbiol (2016) 6:20. 10.3389/fcimb.2016.00020 26904518 PMC4748044

[189] Lamkanfi M Dixit VM. Manipulation of host cell death pathways during microbial infections. Cell Host Microbe (2010) 8:44–54. 10.1016/j.chom.2010.06.007 20638641

[190] Slonchak A Hugo LE Freney ME Hall-Mendelin S Amarilla AA Torres FJ. Zika virus noncoding RNA suppresses apoptosis and is required for virus transmission by mosquitoes. Nat Commun (2020) 11:2205. 10.1038/s41467-020-16086-y 32371874 PMC7200751

[191] Alberdi P Ayllon N Cabezas-Cruz A Bell-Sakyi L Zweygarth E Stuen S. Infection of *Ixodes* spp. tick cells with different *Anaplasma phagocytophilum* isolates induces the inhibition of apoptotic cell death. Ticks Tick Borne Dis (2015) 6:758–67. 10.1016/j.ttbdis.2015.07.001 26183310

[192] Ayllon N Villar M Busby AT Kocan KM Blouin EF Bonzon-Kulichenko E. *Anaplasma phagocytophilum* inhibits apoptosis and promotes cytoskeleton rearrangement for infection of tick cells. Infect Immun (2013) 81:2415–25. 10.1128/IAI.00194-13 PMC3697600 23630955

[193] Ayllon N Villar M Galindo RC Kocan KM Sima R Lopez JA. Systems biology of tissue-specific response to *Anaplasma phagocytophilum* reveals differentiated apoptosis in the tick vector *Ixodes scapularis*. PloS Genet (2015) 11:e1005120. 10.1371/journal.pgen.1005120 25815810 PMC4376793

[194] Rikihisa Y. *Anaplasma phagocytophilum* and *Ehrlichia chaffeensis*: subversive manipulators of host cells. Nat Rev Microbiol (2010) 8:328–39. 10.1038/nrmicro2318 20372158

[195] Martins LA Palmisano G Cortez M Kawahara R de Freitas Balanco JM Fujita A. The intracellular bacterium *Rickettsia rickettsii* exerts an inhibitory effect on the apoptosis of tick cells. Parasit Vectors (2020) 13:603. 10.1186/s13071-020-04477-5 33261663 PMC7706286

[196] Berthelet J Dubrez L. Regulation of apoptosis by inhibitors of apoptosis (IAPs). Cells (2013) 2:163–87. 10.3390/cells2010163 PMC3972657 24709650

[197] Orme M Meier P. Inhibitor of apoptosis proteins in *Drosophila*: gatekeepers of death. Apoptosis (2009) 14:950–60. 10.1007/s10495-009-0358-2 19495985

[198] Gesellchen V Kuttenkeuler D Steckel M Pelte N Boutros M. An RNA interference screen identifies inhibitor of apoptosis protein 2 as a regulator of innate immune signalling in *Drosophila*. EMBO Rep (2005) 6:979–84. 10.1038/sj.embor.7400530 PMC1369191 16170305

[199] Huh JR Foe I Muro I Chen CH Seol JH Yoo SJ. The *Drosophila* inhibitor of apoptosis (IAP) DIAP2 is dispensable for cell survival, required for the innate immune response to gram-negative bacterial infection, and can be negatively regulated by the reaper/hid/grim family of IAP-binding apoptosis inducers. J Biol Chem (2007) 282:2056–68. 10.1074/jbc.M608051200 17068333

[200] Kleino A Valanne S Ulvila J Kallio J Myllymaki H Enwald H. Inhibitor of apoptosis 2 and TAK1-binding protein are components of the *Drosophila* Imd pathway. EMBO J (2005) 24:3423–34. 10.1038/sj.emboj.7600807 PMC1276168 16163390

[201] Leulier F Lhocine N Lemaitre B Meier P. The *Drosophila* inhibitor of apoptosis protein DIAP2 functions in innate immunity and is essential to resist gram-negative bacterial infection. Mol Cell Biol (2006) 26:7821–31. 10.1128/MCB.00548-06 PMC1636742 16894030

[202] Severo MS Choy A Stephens KD Sakhon OS Chen G Chung DW. The E3 ubiquitin ligase XIAP restricts *Anaplasma phagocytophilum* colonization of *Ixodes scapularis* ticks. J Infect Dis (2013) 11:1830–40. 10.1093/infdis/jit380 PMC3814841 23901084

[203] Bonnet SI Binetruy F Hernandez-Jarguin AM Duron O. The tick microbiome: why non-pathogenic microorganisms matter in tick biology and pathogen transmission. Front Cell Infect Microbiol (2017) 7:236. 10.3389/fcimb.2017.00236 28642842 PMC5462901

[204] Narasimhan S Schuijt TJ Abraham NM Rajeevan N Coumou J Graham M. Modulation of the tick gut milieu by a secreted tick protein favors *Borrelia burgdorferi* colonization. Nat Commun (2017) 8:184. 10.1038/s41467-017-00208-0 28775250 PMC5543126

[205] Duron O Binetruy F Noel V Cremaschi J McCoy KD Arnathau C. Evolutionary changes in symbiont community structure in ticks. Mol Ecol (2017) 26:2905–21. 10.1111/mec.14094 28281305

[206] Pavanelo DB Schroder NCH Pin Viso ND Martins LA Malossi CD Galletti M. Comparative analysis of the midgut microbiota of two natural tick vectors of *Rickettsia rickettsii*. Dev Comp Immunol (2020) 106:103606. 10.1016/j.dci.2019.103606 31904432

[207] Barletta A Nascimento-Silva M Talyuli O Oliveira J Pereira L Oliveira P. Microbiota activates IMD pathway and limits Sindbis infection in *Aedes aegypti*. Parasit Vectors (2017) 10:103. 10.1186/s13071-017-2040-9 28231846 PMC5324288

[208] Xiao X Yang L Pang X Zhang R Zhu Y Wang P. A Mesh-Duox pathway regulates homeostasis in the insect gut. Nat Microbiol (2017) 2:17020. 10.1038/nmicrobiol.2017.20 28248301 PMC5332881

[209] Oliveira JH Goncalves RL Lara FA Dias FA Gandara AC Menna-Barreto RF. Blood meal-derived heme decreases ROS levels in the midgut of *Aedes aegypti and* allows proliferation of intestinal microbiota. PloS Pathog (2011) 7:e1001320. 10.1371/journal.ppat.1001320 21445237 PMC3060171

[210] Pang X Xiao X Liu Y Zhang R Liu J Liu Q. Mosquito C-type lectins maintain gut microbiome homeostasis. Nat Microbiol (2016) 1:16023. 10.1038/nmicrobiol.2016.23 PMC4861079 27572642

[211] Caragata E Tikhe C Dimopoulos G. Curious entanglements: interactions between mosquitoes, their microbiota, and arboviruses. Curr Opin Virol (2019) 37:26–36. 10.1016/j.coviro.2019.05.005 31176069 PMC6768729

[212] Moreno-García M Vargas V Ramírez-Bello I Hernández-Martínez G Lanz-Mendoza H. Bacterial exposure at the larval stage induced sexual immune dimorphism and priming in adult *Aedes aegypti* mosquitoes. PloS One (2015) 10:e0133240. 10.1371/journal.pone.0133240 26181517 PMC4504673

[213] Dickson L Jiolle D Minard G Moltini-Conclois I Volant S Ghozlane A. Carryover effects of larval exposure to different environmental bacteria drive adult trait variation in a mosquito vector. Sci Adv (2017) 3:e1700585. 10.1126/sciadv.1700585 28835919 PMC5559213

[214] Moltini-Conclois I Stalinski R Tetreau G Després L Lambrechts L. Larval exposure to the bacterial insecticide Bti enhances Dengue virus susceptibility of adult *Aedes aegypti* mosquitoes. Insects (2018) 9:193. 10.3390/insects9040193 PMC6316598 30558130

[215] Tetreau G Grizard S Patil C Tran F Tran Van V Stalinski R. Bacterial microbiota of *Aedes aegypti* mosquito larvae is altered by intoxication with *Bacillus thuringiensis israelensis*. Parasit Vectors (2018) 11:121. 10.1186/s13071-018-2741-8 29499735 PMC5834902

[216] Zug R Hammerstein P. Still a host of hosts for *Wolbachia*: analysis of recent data suggests that 40% of terrestrial arthropod species are infected. PloS One (2012) 7:e38544. 10.1371/journal.pone.0038544 22685581 PMC3369835

[217] Caragata E Dutra H Moreira L. Exploiting intimate relationships: controlling mosquito-transmitted disease with *Wolbachia*. Trends Parasitol (2016) 32:207–18. 10.1016/j.pt.2015.10.011 26776329

[218] Bian G Xu Y Lu P Xie Y Xi Z. The endosymbiotic bacterium *Wolbachia* induces resistance to Dengue virus in *Aedes aegypti*. PloS Pathog (2010) 6:e1000833. 10.1371/journal.ppat.1000833 20368968 PMC2848556

[219] Hoffmann A Montgomery B Popovici J Iturbe-Ormaetxe I Johnson P Muzzi F. Successful establishment of *Wolbachia* in *Aedes* populations to suppress Dengue transmission. Nature (2011) 476:454–7. 10.1038/nature10356 21866160

[220] Walker T Johnson PH Moreira LA Iturbe-Ormaetxe I Frentiu FD McMeniman CJ. The *w*Mel *Wolbachia* strain blocks Dengue and invades caged *Aedes aegypti* populations. Nature (2011) 476:450–3. 10.1038/nature10355 21866159

[221] Dutra H Dos Santos L Caragata E Silva J Villela D Maciel-de-Freitas R. From lab to field: the influence of urban landscapes on the invasive potential of *Wolbachia* in Brazilian *Aedes aegypti* mosquitoes. PloS Negl Trop Dis (2015) 9:e0003689. 10.1371/journal.pntd.0003689 25905888 PMC4408005

[222] Caragata E Rocha M Pereira T Mansur S Dutra H Moreira L. Pathogen blocking in *Wolbachia*-infected *Aedes aegypti* is not affected by Zika and Dengue virus co-infection. PloS Negl Trop Dis (2019) 13:e0007443. 10.1371/journal.pntd.0007443 31107912 PMC6544317

[223] Andreotti R Perez de Leon AA Dowd SE Guerrero FD Bendele KG Scoles GA. Assessment of bacterial diversity in the cattle tick *Rhipicephalus (Boophilus) microplus* through tag-encoded pyrosequencing. BMC Microbiol (2011) 11:6. 10.1186/1471-2180-11-6 21211038 PMC3025832

[224] Cerutti F Modesto P Rizzo F Cravero A Jurman I Costa S. The microbiota of hematophagous ectoparasites collected from migratory birds. PloS One (2018) 13:e0202270. 10.1371/journal.pone.0202270 30148833 PMC6110481

[225] Estrada-Peña A Cabezas-Cruz A Obregón D. Resistance of tick gut microbiome to anti-tick vaccines, pathogen infection and antimicrobial peptides. Pathogens (2020) 9:309. 10.3390/pathogens9040309 PMC7238099 32331444

[226] Ross BD Hayes B Radey MC Lee X Josek T Bjork J. *Ixodes scapularis* does not harbor a stable midgut microbiome. ISME J (2018) 12:2596–607. 10.1038/s41396-018-0161-6 PMC6194123 29946195

[227] Kurokawa C Lynn GE Pedra JHF Pal U Narasimhan S Fikrig E. Interactions between *Borrelia burgdorferi* and ticks. Nat Rev Microbiol (2020) 18:1–14. 10.1038/s41579-020-0400-5 32651470 PMC7351536

[228] Guizzo MG Neupane S Kucera M Perner J Frantova H da Silva Vaz I. Poor unstable midgut microbiome of hard ticks contrasts with abundant and stable monospecific microbiome in ovaries. Front Cell Infect Microbiol (2020) 10:211. 10.3389/fcimb.2020.00211 32457850 PMC7225584

[229] Guizzo MG Parizi LF Nunes RD Schama R Albano RM Tirloni L. A *Coxiella* mutualist symbiont is essential to the development of *Rhipicephalus microplus*. Sci Rep (2017) 7:17554. 10.1038/s41598-017-17309-x 29242567 PMC5730597

[230] Gall CA Reif KE Scoles GA Mason KL Mousel M Noh SM. The bacterial microbiome of *Dermacentor andersoni* ticks influences pathogen susceptibility. ISME J (2016) 10:1846–55. 10.1038/ismej.2015.266 PMC5029153 26882265

[231] Clayton KA Gall CA Mason KL Scoles GA Brayton KA. The characterization and manipulation of the bacterial microbiome of the Rocky Mountain wood tick, *Dermacentor andersoni*. Parasit Vectors (2015) 8:632. 10.1186/s13071-015-1245-z 26653035 PMC4674957

[232] Labruna MB Ogrzewalska M Martins TF Pinter A Horta MC. Comparative susceptibility of larval stages of *Amblyomma aureolatum*, *Amblyomma cajennense*, and *Rhipicephalus sanguineus* to infection by *Rickettsia rickettsii*. J Med Entomol (2008) 45:1156–9. 10.1603/0022-2585(2008)45[1156:csolso]2.0.co;2 19058642

[233] Martins LA Galletti M Ribeiro JM Fujita A Costa FB Labruna MB. The distinct transcriptional response of the midgut of *Amblyomma sculptum* and *Amblyomma aureolatum* ticks to *Rickettsia rickettsii* correlates to their differences in susceptibility to infection. Front Cell Infect Microbiol (2017) 7:129. 10.3389/fcimb.2017.00129 28503490 PMC5409265

[234] Cooper D Eleftherianos I. Memory and specificity in the insect immune system: current perspectives and future challenges. Front Immunol (2017) 8:539. 10.3389/fimmu.2017.00539 28536580 PMC5422463

